# Dysregulated BMP2 in the Placenta May Contribute to Early-Onset Preeclampsia by Regulating Human Trophoblast Expression of Extracellular Matrix and Adhesion Molecules

**DOI:** 10.3389/fcell.2021.768669

**Published:** 2021-12-14

**Authors:** Yuyin Yi, Hua Zhu, Christian Klausen, Hsun-Ming Chang, Amy M. Inkster, Jefferson Terry, Peter C. K. Leung

**Affiliations:** ^1^ Department of Obstetrics and Gynaecology, BC Children’s Hospital Research Institute, University of British Columbia, Vancouver, BC, Canada; ^2^ Department of Medical Genetics, BC Children’s Hospital Research Institute, University of British Columbia, Vancouver, BC, Canada; ^3^ Department of Pathology and Laboratory Medicine, BC Children’s Hospital Research Institute, University of British Columbia, Vancouver, BC, Canada

**Keywords:** TGF-β superfamily, early-onset preeclampsia, trophoblast, mRNA-seq, BMP2 (bone morphogenetic protein 2), AMIGO

## Abstract

Many pregnancy disorders, including early-onset preeclampsia (EOPE), are associated with defects in placental trophoblast cell invasion and differentiation during early placental development. Bone morphogenetic protein 2 (BMP2) belongs to the TGF-β superfamily and controls various physiological and developmental processes. However, the expression of BMP2 in the placenta and underlying molecular mechanisms of how BMP2 regulates trophoblast function remain unclear. In this study, we analyzed several publicly available microarray and RNA-seq datasets and revealed differences in expression of TGF-β superfamily members between gestational age-matched non-preeclamptic control and EOPE placentas. Importantly, *BMP2* levels were significantly reduced in EOPE placentas compared with controls, and RNAscope *in situ* hybridization further demonstrated *BMP2* expression was disrupted in EOPE placental villi. To explore the molecular mechanisms of BMP2-regulated early trophoblast differentiation, we examined BMP2 expression in first-trimester human placenta and found it to be localized to all subtypes of trophoblasts and the decidua. RNA-seq analysis on control and BMP2-treated primary human trophoblast cells identified 431 differentially expressed genes, including several canonical TGF-β/BMP signaling targets (*BAMBI*, *ID1*, *INHBA*, *IGFBP3*). Gene ontology annotations revealed that differentially expressed genes were involved in cell adhesion and extracellular matrix organization. Furthermore, we identified adhesion molecule with IgG-like domain 2 (AMIGO2) as a novel target for BMP2 that contributed to BMP2-induced trophoblast invasion and endothelial-like tube formation. Overall, our findings provide insight into the molecular processes controlled by BMP2 during early placental development that may contribute to the pathogenesis of EOPE.

## Introduction

Preeclampsia is a pregnancy-specific complication that affects 2–8% of pregnancies and causes a high risk of maternal and fetal morbidity and mortality (Khalil et al., 2016). Depending on the time of onset, preeclampsia can be subclassified into early-onset preeclampsia (EOPE; < 34 weeks gestation) and late-onset preeclampsia (≥34 weeks gestation). EOPE only occurs in 5–20% of preeclampsia cases but is more severe compared to late-onset disease. It is now appreciated that early- and late-onset preeclampsia have different pathophysiologies. Late-onset preeclampsia is associated to a greater degree with maternal constitutive factors or pre-existing susceptibility to vascular damage, and to a lesser extent with defective placentation (Raymond and Peterson, 2011). In contrast, EOPE has been attributed to shallow extravillous trophoblast (EVT) invasion and deficiencies in uterine spiral artery remodeling during the first-trimester of pregnancy that lead to insufficient fetal blood supply (Huppertz, 2008).

The placenta is a temporary organ that connects the fetus to the mother and provides essential supports for maintenance and protection of the developing fetus throughout pregnancy. In early pregnancy, placental cells of epithelial lineage, referred to as trophoblasts, undergo extensive proliferation and differentiation to support formation of a functional placenta. Within the placenta, the villous structure is lined with a multinucleated syncytiotrophoblast (STB) layer generated by fusion of mitotically active mononucleated villous cytotrophoblast (CTB) cells. Surrounded by maternal blood, the STB is the major site for nutrient and gas exchange and synthesis of pregnancy-related hormones (e.g. human chorionic gonadotropin, hCG) (Hamilton and Boyd, 1960; Evain-Brion and Malassine, 2003). In anchoring villi, a population of rapidly proliferating CTBs forms a cell column anchoring the placenta to the maternal uterine wall (Vicovac et al., 1995). CTBs detach from the distal ends of the cell column, gradually exit the cell cycle, and differentiate into extravillous trophoblasts (EVTs) that invade the decidua and maternal vasculature (Pijnenborg et al., 1980). Remodeling of uterine spiral arteries promotes a constant and increased maternal blood flow to the placenta, thereby sustaining the oxygen and nutrient supply for fetal growth throughout gestation (Burton et al., 2010). The process of trophoblast differentiation and invasion are precisely modulated by numerous paracrine and autocrine factors, including cytokines, growth factors, adhesion molecules, and extracellular matrix (ECM) components (Bischof et al., 2000).

In EOPE patients, many factors exhibit aberrant maternal and placental expression leading to trophoblast dysfunction and reduced placental perfusion (Nikuei et al., 2015). One group of prominent factors is the transforming growth factor-β (TGF-β) superfamily of growth factors. More than 40 TGF-β superfamily members have been identified and are largely comprised of the TGF-βs, activins and inhibins, bone morphogenic proteins (BMPs), growth differentiation factors (GDFs), and anti-Müllerian hormone (AMH). The BMP subfamily consists of over 20 members and can be classified into several subgroups based on structural homology, including the BMP2/4, BMP5/6/7/8, BMP9/10, and BMP12/13/14 (GDF5/6/7) groups (Katagiri and Watabe, 2016). Recent studies have demonstrated pro-invasive effects of BMP2 on EVTs *in vitro* (Zhao et al., 2018b), pointing to critical roles for BMPs in trophoblast lineage differentiation and cellular functions. However, to date, there have been no reports comparing the placental expression of BMPs in normal pregnancy or pregnancy complications. Thus, roles of the TGF-β superfamily in placental trophoblast development and in the pathophysiological events of preeclampsia await further elucidation.

## Materials and Methods

### Microarray Gene Expression Analysis

Microarray and RNA-seq datasets from EOPE and gestational age-matched normotensive controls were obtained through the Gene Expression Omnibus (https://www.ncbi.nlm.nih.gov/geo/) under GSE75010 (Leavey et al., 2016), GSE44711 (Blair et al., 2013), and GSE114691 (Awamleh et al., 2019). Microarray data from placentas of differing gestational age was under GSE100051 (Soncin et al., 2018). Differentially expressed genes were tested with a linear model adjusted for fetal sex using limma package (Ritchie et al., 2015) in R version 3.6.1 (R Core Team, 2019). Hierarchical clustering of normalized gene expression data was performed with the hclust function (R Core Team, 2019) (method = “complete”, distance = “Euclidean””). The sigClust2 package (Huang et al., 2015) in R determined if any clusters were significantly different from one another. The pvClust package (Suzuki and Shimodaira, 2006) assessed the stability of the clusters with 1000 bootstraps.

### Patient Recruitment and Tissue Collection

This study was approved by the Research Ethics Board of the University of British Columbia (certificate #H07-01149). First-trimester placental villi and corresponding decidua samples (6–10 weeks) were collected from women undergoing elective surgical termination of pregnancy in the CARE Program at the BC Women’s Hospital & Health Centre. Informed written consent was obtained from participating women. Deidentified term formalin fixed paraffin embedded (FFPE) samples were obtained from placentas submitted for examination between 2014 and 2019 in the BC Children’s Hospital Anatomical Pathology archives. The pre-eclampsia group was defined according to the following clinical features: systolic blood pressure (BP) ≥ 140 mmHg and/or diastolic BP ≥ 90 mmHg after 20 weeks gestation plus proteinuria >300 mg/day or ≥2 + by dipstick. Control samples were derived from otherwise normal preterm multiple gestation deliveries.

### Human Primary Trophoblast and HTR-8/SVneo Immortalized Human Extravillous Trophoblasts Cells

Primary human trophoblast cells were isolated from chorionic villous explants and cultured at 37°C in a 5% CO_2_ air atmosphere as previously described (Irving et al., 1995). Briefly, chorionic villi tips were rinsed in cold Dulbecco’s Modified PBS (Gibco, Thermo Fisher Scientific) and mechanically minced into 1–2 mm fragments. Villous tissue fragments were cultured in Dulbecco’s Minimum Essential Medium (DMEM; Gibco) supplemented with 10% fetal bovine serum (FBS; Gibco), 100  U/ml penicillin, and 100 μg/ml streptomycin for 3–4 days and non-attached fragments were removed. After an additional 10–14 days of culture to allow trophoblast outgrowth, cells were collected from attached fragments by trypsinization.

The HTR-8/SVneo human immortalized extravillous trophoblast cell line was a generous gift from Dr. P. K. Lala (Western University, London, Ontario, Canada) (Graham et al., 1993). Cells were cultured in a 1:1 (v/v) mixture of DMEM/F12 medium (Gibco) supplemented with 10% FBS. Cultures were maintained at 37°C in a humidified atmosphere of 5% CO_2_ in air.

### RNAScope

RNA *in situ* hybridization was performed using RNAscope 2.5 HD Assay-BROWN (Advanced Cell Diagnostics) following the manufacturer’s instructions (Wang et al., 2012). Briefly, formalin-fixed, paraffin-embedded sections were deparaffinized and pretreated with RNAscope Hydrogen Peroxide (room temperature for 10 min), RNAscope Target Retrieval Reagent (95°C for 15 min), and RNAscope Protease Plus Reagent (40°C for 30 min). RNAScope probes targeting BMP2 (Probe-Hs-BMP2; 430641) and negative control (Probe-dapB; 310043) were incubated on sections for 2 h at 40°C and hybridized with RNAscope 2.5 AMP 1-6. RNAscope signal was detected for 10 min at room temperature with a 1:1 mixture of RNAscope 2.5 HD DAB-A and RNAscope 2.5 HD DAB-B. Sections were counterstained with Harris hematoxylin (Sigma-Aldrich) and mounted in a xylene-based mounting medium. Images were captured with an Olympus BX61 light microscope (Olympus) and quantitative assessment of BMP2 mRNA expression was performed utilizing CellProfiler (www.cellprofiler.org). Nuclei were grouped into 5 bins based on the number of dots per nucleus (Bin 0, 0 dots/nucleus; Bin 1, 1–3 dots/nucleus; Bin 2, 4–9 dots/nucleus; Bin 3, 10–15 dots/nucleus; Bin 4, >15 dots/nucleus). H-scores were calculated by totaling the percentage of nuclei in each bin, according to the weighted formula:

$$\begin{array}{l} \text{H}-\text{score}=0\times \left(\%\;of\;nuclei\;in\;bin0\right)+1\times \left(\%\;of\;nuclei\;in\;bin1\right)\\ +2\times \left(\%\;of\;nuclei\;in\;bin2\right)+3\\ \times \left(\%\;of\;nuclei\;in\;bin3\right)+4\times \left(\%\;of\;nuclei\;in\;bin4\right) \end{array}$$

### RNA-Sequencing and Bioinformatic Analysis

Total RNA from human primary trophoblasts was extracted using the RNeasy Mini Kit (Qiagen) according to the manufacturer’s instructions. Sample quality control was performed with the 2100 Bioanalyzer (Agilent Technologies) and qualifying samples (RNA integrity number >7.5) were prepared following the standard protocol for Next Ultra II Stranded mRNA (New England Biolabs). Sequencing was performed on the Illumina NextSeq 500 with paired-end 42 bp × 42 bp reads at the Biomedical Research Centre (University of British Columbia). Reads were aligned with STAR to the UCSC Homo sapiens hg19 (GRCh37) reference sequence (Dobin et al., 2013). Fragments per kilobase of exon per million reads (FPKMs) and differential expression of novel and reference transcripts were calculated using Cufflinks2 (Trapnell et al., 2012). For downstream analyses, genes had to be ≥10 FPKM and ≥1.5-fold change. Gene Ontology (GO) enrichment analysis and KEGG pathway analysis were performed using the enrichGO package (Yu et al., 2012) in R3.6.1. Data have been deposited in NCBI’s Gene Expression Omnibus repository (Edgar et al., 2002) and are accessible through GEO Series accession number GSE172040 with a reviewer token *wdojmqssxbqnvcf*. More detailed information of the materials and methods are provided in the Supplementary Material and Methods.

### Reverse Transcription Quantitative Real-Time PCR

Total RNA from cells was isolated using TRIzol (Invitrogen, Thermo Fisher Scientific) according to the manufacturer’s instructions. cDNA was synthesized using Moloney murine leukemia virus (M-MLV) reverse transcriptase (Promega, Madison, WI). Quantitative real-time PCR was performed with SYBR Green PCR Master Mix (Applied Biosystems, Thermo Fisher Scientific) as per the manufacturer’s instructions on a QuantStudio 3 instrument and data were analyzed with QuantStudio Design and Analysis software (Applied Biosystems). Relative quantification of mRNA levels was performed by the comparative Cq method with GAPDH as the reference gene and using the formula 2^−ΔΔCq^. The primers used in this study are listed in Supplementary Table S1.

### Western Blots

Cells were lysed in lysis buffer (Cell Signaling Technology) supplemented with protease inhibitor cocktail (Sigma). Equal amounts of protein were separated by 10% SDS-PAGE and transferred to PVDF membranes (Bio-Rad). After blocking with 5% non-fat dry milk in Tris-buffered saline for 1 h at room temperature, membranes were incubated at 4°C overnight with anti-AMIGO2 (1:1000, HPA054004, Sigma-Aldrich) or anti-GAPDH (1:2000, sc-365062, Santa Cruz Biotechnology) primary antibodies diluted in TBS with 5% milk. Membranes were then washed and probed for 1 h at room temperature with horseradish peroxidase (HRP)-conjugated secondary. Immunoreactive proteins were detected using the enhanced chemiluminescence kit (Pierce, Thermo Fisher Scientific) and X-ray film.

### Immunohistochemistry and Immunofluorescence

First-trimester human placenta samples (8–10 weeks, *n* = 5) were fixed in 4% formaldehyde at 4°C overnight. Tissues were dehydrated with 70% ethanol, embedded in paraffin, and serially sectioned at 5 µm onto glass slides. Sections were deparaffinized in xylene, rehydrated through graded ethanol, and processed for wet heat-induced antigen retrieval in a steamer for 20 min with a modified citrate buffer (pH6.0; Dako). Endogenous peroxidase activity was quenched with 3% H_2_O_2_ for 10 min at room temperature. After blocking with serum-free protein block (Dako) for 1 h at room temperature, sections were incubated overnight at 4°C with the following primary antibodies: mouse monoclonal anti-human leukocyte antigen-G (HLA-G, 1:100, clone 4H84; ExBio), mouse monoclonal anti-cytokeratin 7 (1:100, RCK105, Santa Cruz Biotechnology), and rabbit polyclonal anti-AMIGO2 (1:100, HPA054004, Sigma-Aldrich). Immunoreactivity was detected with the LSAB 2 System-HRP kit (K0690, Dako) and 3, 3′-diaminobenzidine (DAB) chromogen solution (Dako). Slides were counterstained with Harris hematoxylin (Sigma-Aldrich), dehydrated through graded ethanol to xylene, and mounted in a xylene-based mounting medium.

For immunofluorescence staining, cells were cultured on coverslips, fixed with 4% paraformaldehyde for 10 min at room temperature, and permeabilized with 0.1% Triton X-100 (Sigma) for 5 min. Coverslips were blocked with 5% BSA in PBS for 1 h at room temperature and then incubated with primary antibodies overnight at 4°C. Coverslips were incubated for 1 h at room temperature with Alexa Fluor goat anti-mouse 594 conjugated secondary antibodies (Life Technologies) and mounted using ProLong Gold mounting media containing DAPI (Life Technologies). Images were captured with an Olympus BX61 light microscope (Olympus Canada, Richmond Hill, ON) and analyzed using ImageJ software.

### Small Interfering RNA Transfection

To knockdown endogenous AMIGO2, HTR-8/SVneo cells (1.5 × 10^5^/well) or primary trophoblasts (3 × 10^5^/well) were seeded in a 6-well plate 24 h before transfection. Cells were transfected with 50 nM ONTARGETplus SMARTpool siRNA (Dharmacon, Lafayette, CO) targeting AMIGO2 using 5 µl Lipofectamine RNAiMAX (Invitrogen, Thermo Fisher Scientific). The siCONTROL non-targeting pool (Dharmacon) was used as the transfection control. Cells were then cultured for 48 h and harvested for assessment of knockdown efficiency (Western blot) and functional analyses (invasion and endothelial-like tube formation).

### Invasion and Endothelial-like Tube Formation Assays

For invasion assays, transwell cell culture inserts (8-μm pore size, 24 wells, BD Biosciences) were coated with 1 mg/ml of growth factor-reduced Matrigel (BD Biosciences). Treated cells (1 × 10^5^ cells/insert) in medium supplemented with 0.1% FBS were incubated for 48 h against a gradient of 10% FBS. Non-invading cells were removed with a cotton swab from the upper side of the membrane. Cells that penetrated the membrane were fixed with cold methanol and stained with crystal violet (0.5%, Sigma) for 30 min and subsequently washed thoroughly with tap water.

For endothelial-like tube formation assays, individual wells of a 96-well plate were each coated with 30 μl growth factor-reduced Matrigel (BD Biosciences, 5 mg/ml). Coated wells were seeded with 4 × 10^4^ HTR-8/SVneo cells suspended in 100 μl DMEM/F12 medium supplemented with 0.1% FBS and incubated at 37°C for 24 h. Digital images were taken with a light microscope and data was analyzed with Image-J software.

### Statistical Analysis

Results are presented as the mean ± SEM from a minimum of three independent experiments unless otherwise indicated. Student’s *t* test was used for comparisons between two groups. Multiple group comparisons were analyzed by one-way analysis of variance (ANOVA) followed by Tukey’s test using GraphPad Prism version 7.00 for Windows (GraphPad Software, La Jolla California United States, www.graphpad.com). Means were considered significantly different from each other if *p* < 0.05.

## Results

### TGF-β Pathway-Associated Genes Are Differentially Expressed in Placentas From Healthy and Early-Onset Preeclampsia Pregnancies

**FIGURE 1:**
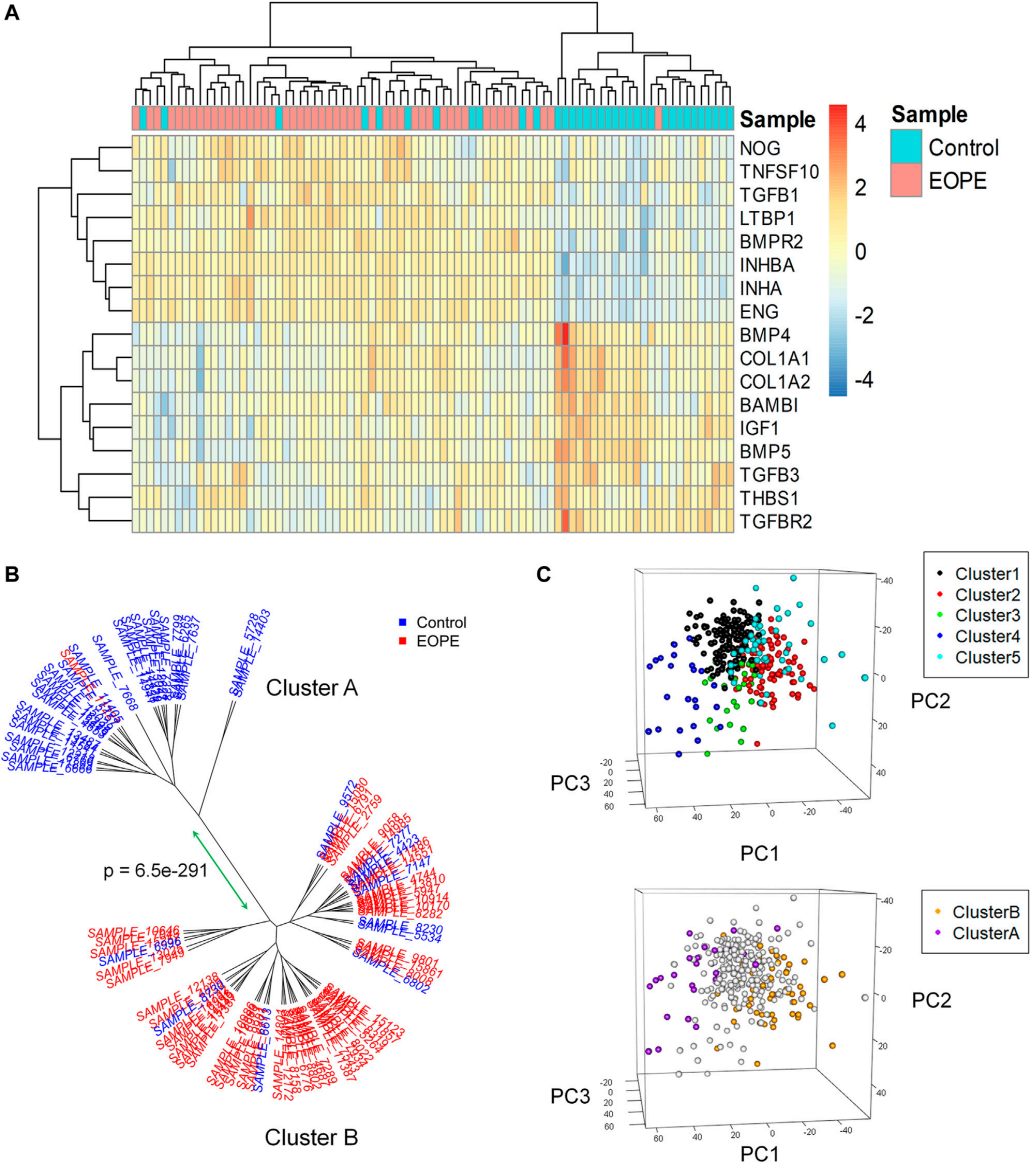
Microarray analysis of TGF-β pathway-associated gene expression in healthy and EOPE placentas. **(A)**, Heatmap of 17 differentially expressed TGF-β pathway genes from GSE75010 whole-genome mRNA microarray analysis between EOPE (*n* = 49) and matched normal control placentas (*n* = 35). **(B)**, Hierarchical clustering of patients in the GSE75010 dataset using 17 differentially expressed genes associated with the TGF-β pathway and the results were plotted as phylogenetic trees. Samples plotting closer together demonstrated more similar gene expression features, with tips colored based on clinical outcome. The sigClust2 and pvCluster packages in R were used to determine stable and significantly different clusters. *p* values signify clusters are significantly different from one another. **(C)**, Principal component analysis (PCA) plots for the visualization. Upper panel was modified from original PCA plot in prior gene expression study (Leavey et al., 2016) and showed the 5 Clusters of samples based on expression of 3663 genes. Bottom panel: Two major clusters (Cluster A and Cluster B) identified in current study corresponded with original Cluster 1/4 and Cluster 2

Microarray data from 157 placenta samples was obtained from GSE75010(Leavey et al., 2016). EOPE (diagnosis <34 weeks) tends to be more severe and associated with the defects in early placental development and trophoblast function, whereas late-onset preeclampsia (≥34 weeks) may arise from a maternal genetic predisposition to cardiovascular and metabolic disease (Burton et al., 2019). Thus, we filtered to cases delivered on or before 34 weeks of gestation, as this is implicitly EOPE (*n* = 49). Patients with preterm labour and no other complications before 34 weeks of gestation were included as controls (*n* = 35). We first investigated the association between EOPE and 84 TGF-β pathway-associated genes encoding TGF-β superfamily ligands, receptors, SMADs, and other relevant proteins. Differential expression analysis was conducted focused on these 84 candidates and identified 17 TGF-β pathway genes that were significantly altered in the EOPE placentas (adjusted *p* < 0.05, 1.2-fold change cut-off). Among them, 8 genes were upregulated and 9 genes were downregulated in EOPE compared with the matched normal placental tissues (Figure 1A and Supplementary Table S2). We applied hierarchical clustering to all samples on the expression values of the 17 differentially expressed TGF-β pathway genes. Unsurprisingly, samples were clustered into two stable and statistically different clusters. Cluster A included almost all preterm control samples whereas Cluster B contained most EOPE samples, suggesting differentially expressed TGF-β pathway genes successfully distinguished EOPE from gestational age-matched non-preeclamptic controls (Figure 1B). Additionally, we confirmed that maternal ethnicity and fetal sex had no significant impact on the bioinformatic clustering of the study (Table 1). Previous meta-analyses of placental gene expression using the same cohort have identified 5 distinct molecular subclasses (Leavey et al., 2016), including control pregnancies or maternal preeclampsia patients with similar placental gene expression profiles (Cluster 1); “canonical” preeclampsia with placental dysfunction markers (Cluster 2); “immunological” preeclampsia patients associated with maternal-placental incompatibility (Cluster 3); the preterm control samples (Cluster 4); and a Cluster 5 with chromosomal abnormalities. We sought to further compare the clusters identified by differentially expressed TGF-β pathway genes with the clusters in the meta-analyses. As shown in Figure 1C, 24 out of 26 Cluster A samples were in Cluster 1 or 4 reported in the previous study (Leavey et al., 2016), implying Cluster A represents the preterm control with placental gene profile similar to healthy pregnancies. In contrast, 43 out of 58 Cluster B samples corresponded with Cluster 2 samples, the “canonical” preeclampsia subclass that likely arises from a placental origin. Leavey *et al.* has reported that the preeclampsia disease appears to be more severe in Cluster 2 and more frequently associated with delivered preterm and coexisting diagnoses of IUGR. Interestingly, our analysis showed that all non-EOPE IUGR cases cluster with the Cluster B containing most EOPE cases (Table 1 and Supplementary Figure S1), although we observed no statistically significant distinctions within Cluster B in the diagnosis with coexisting IUGR.

**TABLE 1:** Clinical information Across Clusters (generated from GSE57010).

	Cluster A (*n* = 26)	Cluster B (*n* = 58)
Clinical Characteristic, continuous attribute Mean (SD)		
Maternal age^a^ (years)	31.1 (5.6) (*n* = 25)	33.3 (6.0) (*n* = 58)
BMI^a^ (kg/m2)	24.24 (5.26)* (*n* = 23)	27.92 (5.95) (*n* = 46)
Maximum systolic blood pressure (mmHg)	125 (15)*	173 (18)
Maximum diastolic blood pressure (mmHg)	78 (11)*	108 (11)
Gestational Age at delivery (weeks)	29 (3)	30 (2)
Newborn weight z-score	0.37 (0.78)*	−1.27 (0.77)
Placental weight z-score	0.33 (1.15)*	−1.24 (0.81)
Clinical Characteristic, categorical attribute Percentage (n/N)		
Maternal ethnicity^a^		
Asian	7.7% (2/26)	17.2% (10/58)
Black	7.7% (2/26)	29.3% (17/58)
Caucasian	69.2% (18/26)	44.8% (26/58)
South Asian	7.7% (2/26)	5.2% (3/58)
Other	7.7% (2/26)	3.4% (2/58)
Preeclampsia diagnosis (Yes)	3.8% (1/26)*	82.8% (48/58)
IUGR diagnosis (Yes)	0% (0/26)*	48.2% (28/58)
Mode of delivery (C-section)	50.0% (13/26)*	96.6% (56/58)
Infant sex (Female)	38.5% (10/26)	55.2% (32/58)

a Information was not available for all patients. *p* value was calculated using Fisher exact tests for categorical variables and Mann-Whitney U tests for continuous variables.

* *p*-value < 0.05.

Among the TGF-β superfamily ligands and receptors, TGFB1, INHBA, INHA, and BMPR2 were significantly upregulated in EOPE, whereas BMP4, BMP5, TGFB3, and TGFBR2 were significantly downregulated, although the differences between groups were small (Supplementary Table S2 and Supplementary Figure S2). Although some studies have shown that plasma levels of TGF-β1 and TGF-β2 are elevated in patients with preeclampsia (Djurovic et al., 1997; Shaarawy et al., 2001; Peracoli et al., 2008), others have not found any differences in serum or placental levels of TGF-βs (Lyall et al., 2001; Perucci et al., 2014). Interestingly, our analysis showed that BMP family ligands – *BMP4*, *BMP5* – were decreased in EOPE. However, the importance of the BMP family in trophoblast differentiation and function during early pregnancy and their relevance to the pathogenesis of placenta-related disorders remains unclear.

### Bone Morphogenetic Protein Family Ligands Are Dysregulated in Early-Onset Preeclampsia Placentas

**FIGURE 2:**
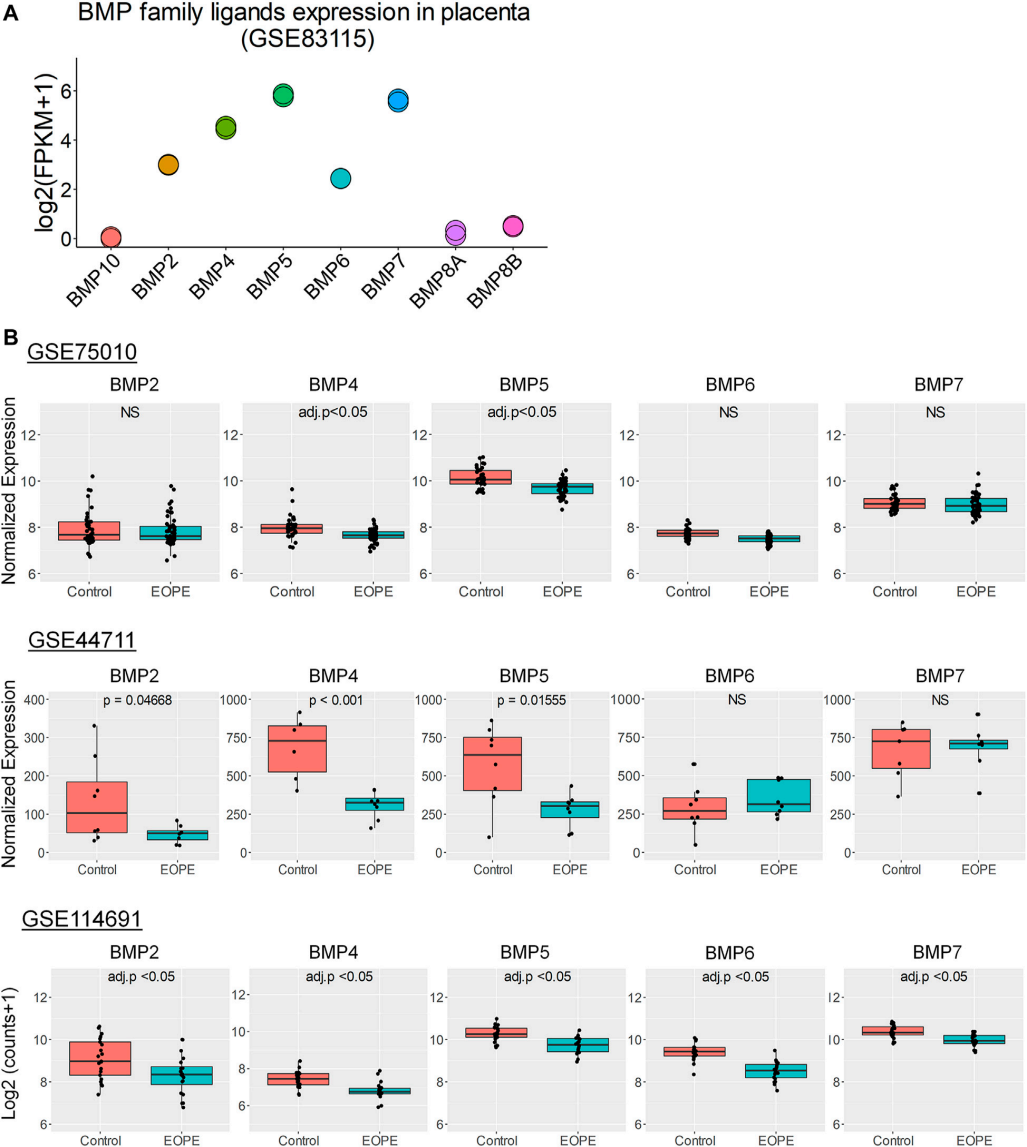
BMP family ligands expression in non-preeclamptic and EOPE placentas. **(A)**, RNA levels of BMP family ligands in the human placenta. Data was generated from GSE83115. **(B)**, Expression levels of BMP family ligands in placental tissues from EOPE *vs.* matched controls. Data were generated from microarray datasets GSE75010 (Control, *n* = 35; EOPE, *n* = 49) and GSE44711 (Control, *n* = 8; EOPE, *n* = 8) as well as RNA-seq dataset GSE114691 (Control, *n* = 21; EOPE, *n* = 20). Expression values were represented as log2 expression microarray data or log2 (counts+1) for RNA-seq data.

To further investigate the clinical significance of BMP family ligands in EOPE, we focused our subsequent analyses on five ligands with relatively high expression in the human placenta; BMP2, BMP4, BMP5, BMP6 and BMP7 (Figure 2A) (Zhu et al., 2016). In addition, we introduced another two datasets which were analyzed in parallel with GSE75010 (Control, *n* = 35; EOPE, *n* = 49) – a microarray dataset GSE44711(Blair et al., 2013) with placenta samples from EOPE pregnancies (*n* = 8) and gestational age-matched control pregnancies (*n* = 8), and an RNA-seq dataset (GSE114691(Awamleh et al., 2019)) generated from human EOPE placentas (*n* = 20) and matched control samples (*n* = 21). As shown in Figure 2B, BMP4 and BMP5 showed a significant downregulation in the placentas from patients with EOPE compared with controls in all three datasets. In addition, analyses from GSE44711 and GSE114691 datasets showed that BMP2 was significantly downregulated in EOPE placentas compared with controls. Of note, BMP2 showed a decreased expression in EOPE placentas in GSE75010, but this difference was not significant (Figure 2B). In contrast, we only observed a significant but relatively small decrease of BMP6 and BMP7 in EOPE placentas from the GSE114691 RNA-seq dataset. Together these analyses suggest the downregulation of BMP2, BMP4 and BMP5 might be associated with EOPE.

**FIGURE 3:**
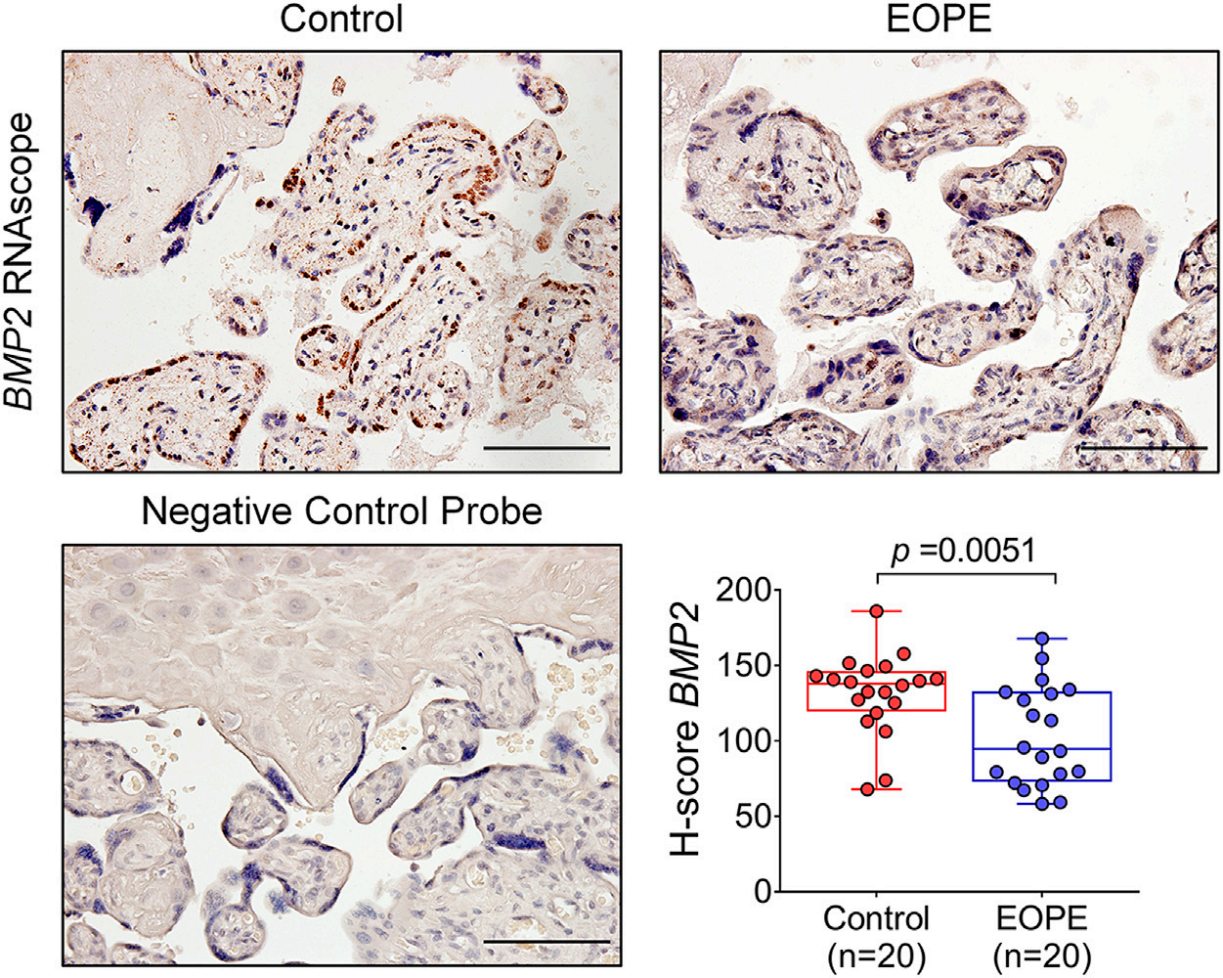
BMP2 is aberrantly expressed in EOPE placenta. Representative image showing *in situ* localization of *BMP2* mRNA transcript (brown) in control and EOPE placentas (200×). *Bottom left*; Negative control probe (DapB) as a negative control for the RNAscope *in situ* hybridization assay (200×). *Bottom right*; Box and whisker plot of *BMP2* H-score (the weighted number of dots/cell, see Materials and Methods) in control and EOPE placentas (*p* = 0.0051 by Students’*t* test). Scale bars = 100 µm.

Our previous work has suggested that BMP2 contributes to the regulation of invasive trophoblast differentiation and spiral artery remodelling (Zhao et al., 2018a; Zhao et al., 2018b; Zhao et al., 2020a; Zhao J. et al., 2020b). Therefore, we next sought to confirm the dysregulation of placental BMP2 expression in preeclampsia samples. Lacking a BMP2 antibody with appropriate specificity, we used RNAscope *in situ* hybridization to examine the localization of *BMP2* mRNA. A retrospective cohort study was conducted to compare the expression and localization of BMP2 in placentas from EOPE (*n* = 20) and non-preeclampsia preterm controls (*n* = 20). The expression of *BMP2* in term placental villi was detected in the STB layer and some villous stromal cells. Importantly, the BMP2 mRNA level (H-score) significantly decreased in the EOPE group compared with the gestational age-matched control group (Figure 3 and Supplementary Figure S3). The clinical characteristics of the preterm control pregnancies and EOPE patients are shown in Table 2. The decreased BMP2 levels in the EOPE placenta link the critical role of BMP2 in early trophoblast development to the pathogenesis of EOPE.

**TABLE 2:** Clinical information of EOPE RNAscope study.

	Control (*n* = 20)	EOPE (*n* = 20)
Clinical Characteristic, continuous attribute Mean (SD)		
Maternal age (years)	32.6 (4.37)	34.0 (5.35)
Maximum systolic blood pressure (mmHg)^a^	NR	169 (16) (*n* = 18)
Maximum diastolic blood pressure (mmHg)^a^	NR	106 (9) (*n* = 18)
Gestational Age at delivery (weeks)	29 (3)	29 (2)
Clinical Characteristic, categorical attribute Percentage (n/N)		
Infant sex (Female)^a^	42.11% (8/19)	45% (9/20)

a Information was not available for all patients. NR: No record

*p* value was calculated using Fisher exact tests for categorical variables and Kruskal-Wallis tests for continuous variables, followed by Bonferroni correction for multiple comparison testing. *p-value < 0.05.

### Localization of Bone Morphogenetic Protein 2 in Human First-Trimester Placental Villi

**FIGURE 4:**
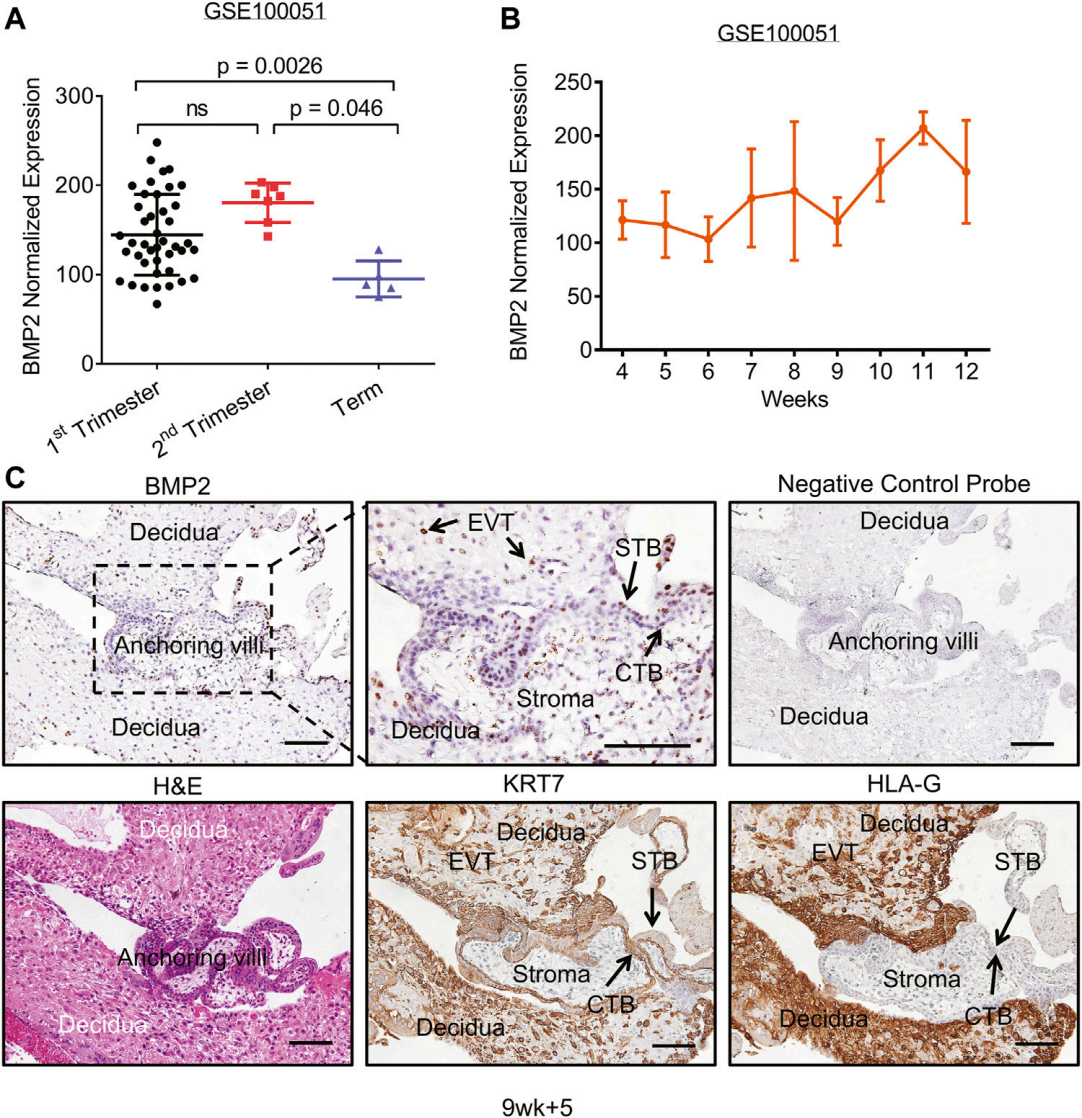
BMP2 expression and localization at the early human maternal-fetal interface. **(A,B)** Microarray analysis of BMP2 levels in placentas from GSE100051. **(A)**, *BMP2* levels in placentas across gestation (1^st^ trimester, *n* = 42; 2^nd^ trimester, *n* = 7; term, *n* = 5). Results are expressed as the mean ± SD and statistical analysis was performed using the Kruskal-Wallis test with Dunn’s correction. **(B)**, Microarray analysis shows an increase in *BMP2* levels in human placentas during the first trimester. *n* = 5 for each gestational age indicated, except 4 weeks gestation (*n* = 3) and 8 weeks gestation (*n* = 4). **(C)**, *Top left*: Representative image showing *in situ* localization *of BMP2* mRNA transcript (brown) within a first-trimester placental villus (9 weeks +5 days gestation; 100×). *Top centre*: Magnification of the boxed area in the top left panel (200×). *Top right*: Negative control probe (DapB) incubation of a serial section as a negative control for the RNAscope *in situ* hybridization assay (100×). *Bottom panels*: Hematoxylin and eosin (H&E) staining **(*left*)** as well as immunohistochemical staining for cytokeratin 7 (KRT7; *centre*) and human leukocyte antigen-G (HLA-G; ***right***) was performed on serial sections of the same placental villi (100×). CTB, cytotrophoblast; STB, syncytiotrophoblast; EVT: extravillous trophoblast. Scale bars = 100 µm.

To examine the expression of BMP2 in the human placenta throughout pregnancy, we re-analyzed another microarray-based transcriptome dataset comprised of normal human placenta samples from different gestational age time-points (4–39 weeks gestation, *n* = 54, GSE100051(Soncin et al., 2018)). As shown in Figure 4A, *BMP2* transcript was expressed in placentas across all three trimesters, with significantly higher levels in the first- and second-trimesters than at term. Further analysis of the first-trimester showed an increasing trend in *BMP2* levels, peaking at 10–12 weeks gestation (Figure 4B). Despite the increasing evidence indicating the importance of BMP2 in placental trophoblast functions, the exact expression pattern and localization of BMP2 in first-trimester human placenta has yet to be described. Next, we used *in situ* hybridization to examine the localization of *BMP2* mRNA in first-trimester human placental tissues (*n* = 5; 8–10 weeks gestation). To identify different subtypes of trophoblast cells, we also performed immunohistochemistry with antibodies against the pan-trophoblast marker cytokeratin 7 (KRT7) and the EVT specific marker HLA-G. RNAscope *in situ* hybridization showed specific and intense *BMP2* mRNA localization to cells within the stroma of placental villi as well as to CTBs, STBs and EVTs (Figure 4C and Supplementary Figure S4). Notably, *BMP2* mRNA was localized to EVTs within the anchoring columns and decidua as indicated by positive staining for the HLA-G antibody (Figure 4C). *BMP2* mRNA was also detected in maternal decidua cells whereas no specific positive signal was detected in serial sections incubated with the negative control probe (Figure 4C).

### Bone Morphogenetic Protein 2 Orchestrates a Gene Network of Cell Adhesion and Extracellular Matrix Molecules in Primary Human Trophoblasts

**FIGURE 5:**
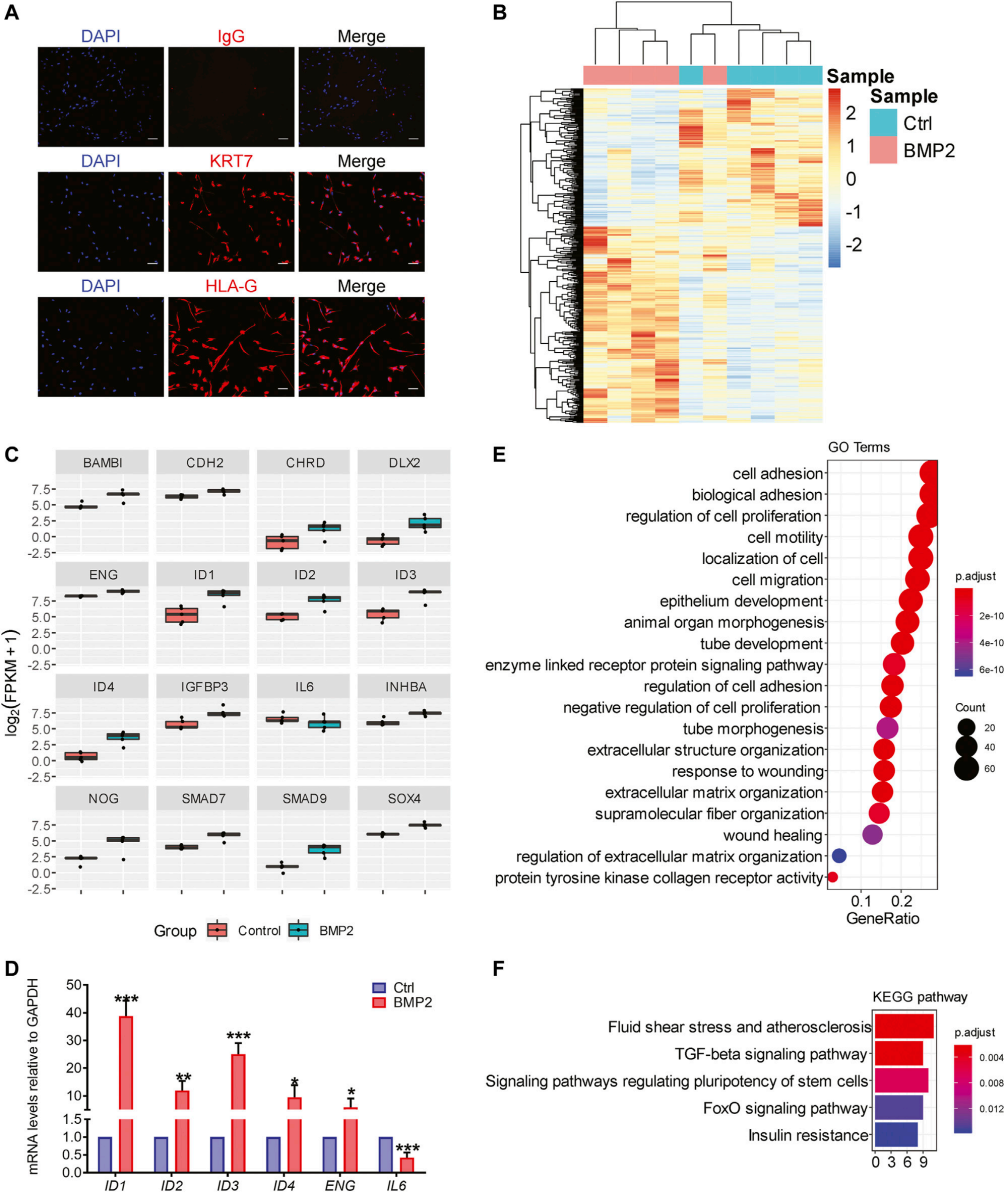
Comparison of differentially expressed genes in human primary trophoblasts treated with or without BMP2. **(A)**. Purified primary human trophoblast cells were immunofluorescently stained with antibodies directed against cytokeratin 7 (KRT7) and human leukocyte antigen-G (HLA-G), and nuclei were stained with DAPI (200 × magnification, scale bars = 100 µm). **(B)**, Hierarchical clustering of control and BMP2 treated trophoblasts using a total of 431 differentially expressed genes from mRNA-seq analysis (row *Z*-score). **(C)**, RNA-seq data of differentially expressed BMP signaling targets in primary trophoblasts treated with or without 25 ng/ml BMP2 (FPKM, fragments per kilobase of exon per million reads). False discovery rate values for all the genes presented were <0.05 from CuffDiff2. **(D)**, RT-qPCR validation of the differential expression of BMP signaling target genes in primary trophoblasts treated with or without 25 ng/ml BMP2, *n* = 5. Data were normalized to *GAPDH* and values represent the mean ± SD. *p < 0.05, **p < 0.01, ***p < 0.001 from Student’s *t* test. **(E)**, Gene Ontology (GO) analysis of differentially expressed genes indicating the top 20 enriched GO terms in BMP2 treated primary trophoblasts. **(F)**, Top 5 KEGG pathways enriched in BMP2 treated primary trophoblasts.

Primary human trophoblasts were isolated from first-trimester placental villi as previously described (Irving et al., 1995). To confirm the characteristics of these primary human trophoblasts, cells were immunofluorescently stained with KRT7 and HLA-G and assessed microscopically to confirm purity of invasive trophoblasts >90% (Figure 5A). To gain mechanistic insight into how BMP2 modulates trophoblast development and function, RNA-seq was used to analyze global gene expression. In this study, primary trophoblast cells from five first-trimester placentas (*n* = 5) were treated with vehicle control or 25 ng/ml BMP2 for 24 h as previously described (Zhao et al., 2018b). RNA-seq analysis identified 431 differentially expressed genes between control and BMP2 treated cells (253 genes upregulated, 178 genes downregulated; false discovery rate <0.05; Supplementary Table S3). A heat-map highlighting patterns of differentially expressed genes across control and BMP2 treated trophoblasts is shown in Figure 5B. RNA-seq analysis identified several canonical TGF-β/BMP signaling targets that were differentially expressed between control and BMP2-treated trophoblasts, including several that have previously been identified as critical for the regulation of human trophoblast invasion and differentiation by BMP2 (*BAMBI*, *CDH2*, *ID1*, *INHBA*, *IGFBP3*; Figure 5C) (Zhao et al., 2018a; Zhao et al., 2018b; Zhao et al., 2020a; Zhao et al., 2020b). RT-qPCR was used to validate the differential expression of a subset of TGF-β/BMP2 target genes (*ID1, ID2, ID3, ID4, ENG* and *IL6*; Figure 5D). Gene Ontology (GO) analysis revealed that BMP2-regulated genes are involved in cell adhesion, cell migration, cell proliferation and ECM organization (Figure 5E and Supplementary Table S4). Unsurprisingly, KEGG pathway analysis demonstrated that total differentially expressed genes were enriched in TGF-β signaling and pluripotency stem cell pathways (Figure 5F). Interestingly, BMP2 also regulates a set of genes enriched in FoxO signaling, insulin resistance, and fluid shear stress and atherosclerosis, suggesting potential links to trophoblast metabolic regulation and trophoblast-endothelial cell remodelling (Figure 5F).

**FIGURE 6:**
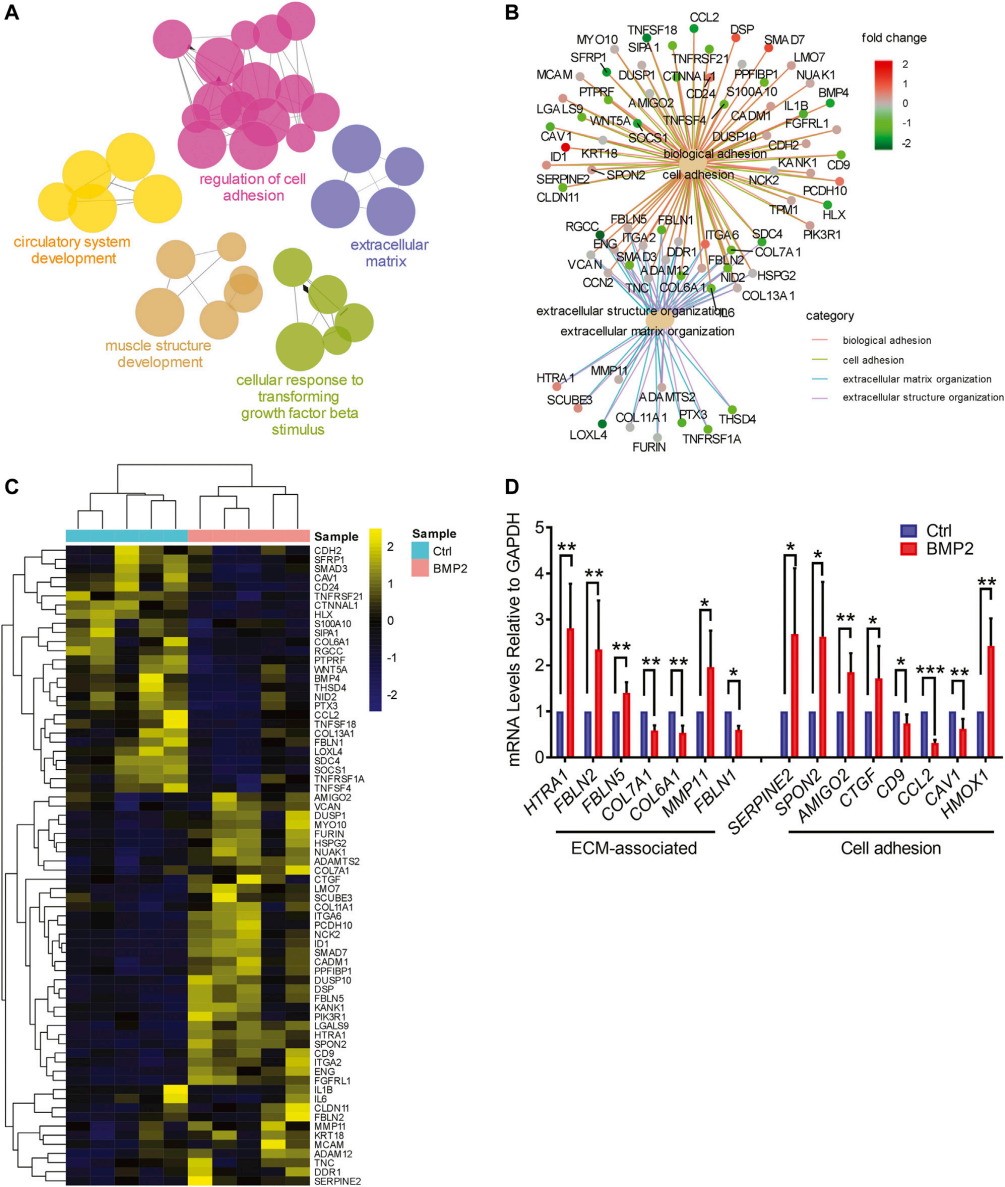
BMP2 regulates the expression of cell adhesion and ECM-associated genes in primary human placental trophoblasts. **(A)**, Functional clustering map for genes differentially expressed in control and BMP2 treated trophoblasts. The map was generated using Cytoscape with ClueGo plug-in and each node represents a pathway or GO term. Links between nodes represent the genes shared by both pathways (*p* cutoff 0.01, Kappa score threshold 0.5). Clusters were manually drawn and annotated. Groupings with less than 4 connections were excluded from the final list of networks. **(B)**, Gene interaction networks of differentially expressed genes within the top 4 enriched GO terms (biological adhesion, cell adhesion, extracellular matrix organization, and extracellular structure organization). Each small dot represents a gene and large dots represent GO terms. Links indicate the association of each gene with the GO terms. Log-transformed fold change for each gene is shown by a colored scale. **(C)**, Relative expression levels (row *Z*-score) of significantly altered genes belonging to the cell-adhesion and ECM-associated categories from RNA-seq data. **(D)**, RT-qPCR validation of differential expression of cell adhesion and ECM-associated genes in primary trophoblasts treated with or without 25 ng/ml BMP2, *n* = 5. Data were normalized to *GAPDH* and values represent the mean ± SD. *p < 0.05, **p < 0.01, ***p < 0.001 from Student’s *t* test.

To verify the functional connectivity of differentially expressed genes, we performed functional annotation clustering using Cytoscape software with the ClueGo plugin. Network mapping identified regulation of cell adhesion and ECM-associated processes as the most enriched clusters, but also showed enrichment of pathways related to circulatory system development, muscle structure development, and cellular response to TGF-β stimulation (Figure 6A). Genes associated with cellular adhesion included *CDH2*, *SERPINE2*, *SPON2*, *CD9*, *ID1*, *AMIGO2*, *WNT5A*, *CCN2*, *CCL2*, *CAV1* (Figures 6B,C). ECM-associated genes regulated by BMP2 included ECM components (*COL6A1, COL7A1, COL11A1, COL13A1*, *FBLN1*, *FBLN2*, *FBLN5*), ECM receptors (*ITGA2*, *ITGA6*, *DDR1*), and proteinases (*ADAM12*, *ADAMTS2*, *MMP11*; Figures 6B,C). RT-qPCR was used to validate the regulation of a subset of these cell adhesion and ECM-associated genes by BMP2 (Figure 6D). Our RNA-seq analysis provides strong evidence that BMP2 regulates gene programmes and processes that are critical for cell adhesion and cell-ECM interaction, thereby promoting trophoblast differentiation along the extravillous invasive pathway.

### Identification of Adhesion Molecule With IgG-Like Domain 2 as a Novel Mediator Required for the Pro-Invasive Effects of Bone Morphogenetic Protein 2 on Human Trophoblast Cell Invasion

**FIGURE 7:**
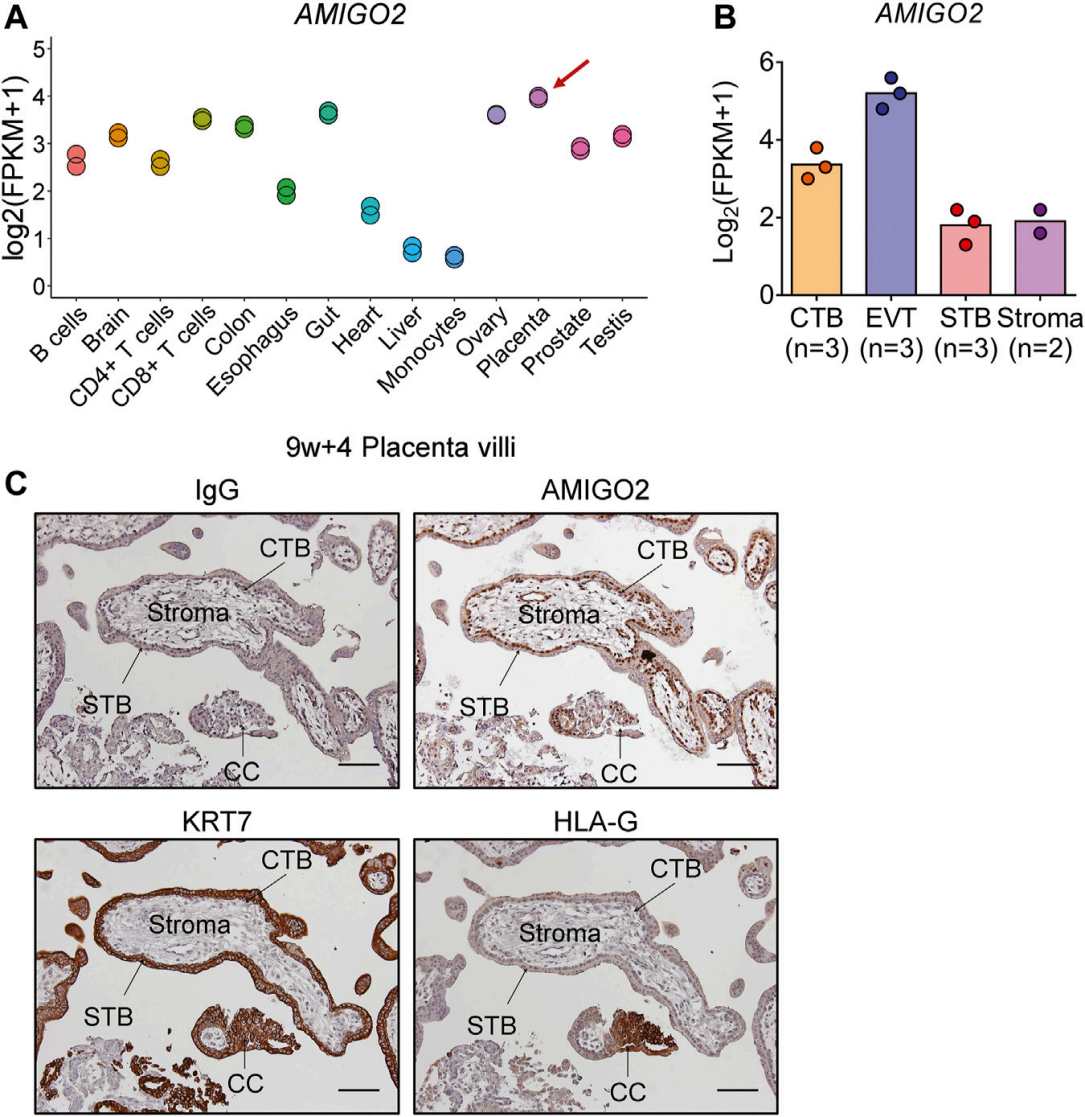
AMIGO2 expression and localization in human placenta. **(A)**, Highest RNA levels of *AMIGO2* detected in placenta among the human organs and tissues. Data was generated from GSE83115. **(B)**, The RNA-seq data of *AMIGO2* levels in primary CTB (*n* = 3), EVT (*n* = 3), STB (*n* = 3), and placenta-derived stromal cells (*n* = 2). Data were generate from Okae H *et al.*,and presented as log_2_(FPKM+1) (Okae et al., 2018). **(C)**, Representative immunohistochemical image showing AMIGO2 localization (brown) within a first-trimester placental villus (9 weeks + 4 days gestation; 100 × magnification, scale bar = 100 µm). Cytokeratin 7 (KRT7; centre) and human leukocyte antigen-G (HLA-G; right) was performed on serial sections of the same placental villi. CTB, cytotrophoblast; SCT, syncytiotrophoblast; EVT, extravillous trophoblast; CC, cell column.

**FIGURE 8:**
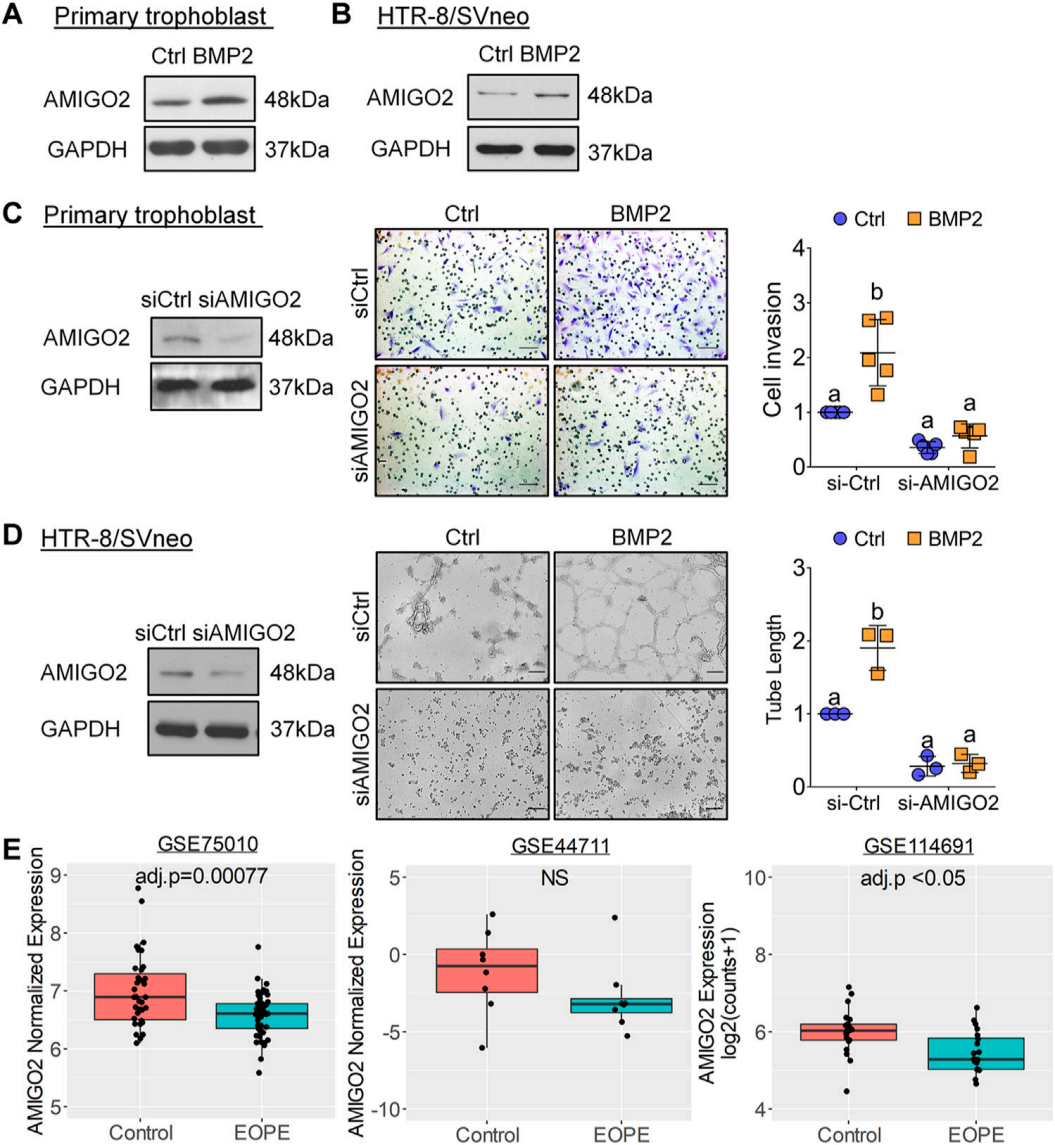
AMIGO2 mediates BMP2-induced human trophoblast invasion and endothelial-like tube formation. **(A,B)**, Representative Western blot images showing the effect of exogenous BMP2 treatment on AMIGO2 protein levels in primary human trophoblasts (*n* = 5) and HTR-8/SVneo cells (*n* = 3). **(C,D)**, Cells were transfected for 48 h with non-targeting control siRNA (siCtrl) or siRNA targeting *AMIGO2* (siAMIGO2) prior to treatment with vehicle (Ctrl) or 25 ng/ml BMP2. AMIGO2 knockdown was confirmed by Western blot **(*left panels*)**. **(C)**, Representative images from Matrigel-coated transwell invasion assays examining the effect of AMIGO2 knockdown on BMP2-induced invasion of primary human trophoblasts (*n* = 5; ***centre panel***). Summarized quantitative results are expressed as the mean ± SD of five independent experiments and values without common letters are significantly different (*p* < 0.05; ***right panel***). **(D)**, Representative images of the effect of AMIGO2 knockdown on BMP2-induced endothelial-like tube formation in HTR-8/SVneo cells (*n* = 3; ***centre panel***). Summarized quantitative results are expressed as the mean ± SD of three independent experiments and values without common letters are significantly different (*p* < 0.05; *right panel*). **(E)**, *AMIGO2* expression is downregulated in EOPE placentas compared with non-preeclamptic placenta tissues. Data were generated from microarray datasets GSE75010 (Control, *n* = 35; EOPE, *n* = 49) and GSE44711 (Control, *n* = 8; EOPE, *n* = 8) as well as RNA-seq dataset GSE114691 (Control, *n* = 21; EOPE, *n* = 20).

Among the transcriptional targets of BMP2 in primary human trophoblasts, we identified AMIGO2 as a novel adhesion molecule associated with cell adhesion and ECM formation (Rabenau et al., 2004) based on its high expression in placenta among human organs and tissues (Figure 7A). In addition, analysis of RNA-seq data of first-trimester human CTB, EVT, STB, and placenta-derived stromal cells (Okae et al., 2018) found that AMIGO2 expression was highest in EVTs (Figure 7B). Further immunohistochemical analysis of first-trimester placentas showed strong expression of AMIGO2 in the cytoplasm and nucleus of villous CTBs and EVTs within anchoring columns as well as decidua cells, whereas expression was weak in STBs (Figure 7C and Supplementary Figure S5). In both RNA-seq and RT-qPCR analyses, *AMIGO2* expression was elevated by BMP2 treatment in primary human trophoblasts (Figures 6C,D). Western blot results confirmed the upregulation of AMIGO2 protein levels following treatment of primary and immortalized human trophoblasts HTR-8/SVneo with BMP2 (Figures 8A,B). Together with our demonstration of AMIGO2 expression in trophoblasts, we hypothesized that AMIGO2 may play a role in regulating trophoblast invasiveness. To study the function of AMIGO2 in human trophoblasts, we used Matrigel-coated transwell assay to model the process of trophoblast invasion through ECM during early placental development. As shown in Figure 8C, siRNA-mediated knockdown of AMIGO2 significantly reduced primary human trophoblast cell invasion, indicating endogenous AMIGO2 is required for trophoblast invasion. We next examined the involvement of AMIGO2 in BMP2-induced trophoblast invasion. Consistent with our previous studies (Zhao et al., 2018b), treatment with BMP2 significantly increased primary human trophoblasts cell invasion and, importantly, these effects were attenuated by AMIGO2 knockdown (Figure 8C). BMP2 has also been shown to promote endothelial-like tube formation of HTR-8/SVneo cells (Zhao et al., 2020a). We showed that knockdown of AMIGO2 dramatically impaired basal and BMP2-mediated endothelial-like tube formation in trophoblastic HTR-8/SVneo cells (Figure 8D and Supplementary Figure S6). Taken together, these results suggest that AMIGO2 contributes to BMP2-induced invasive and endovascular differentiation of human trophoblasts during early placental development. Analysis of microarray datasets showed that *AMIGO2* levels were significantly reduced in placenta samples from EOPE patients compared with gestational age-matched non-preeclamptic controls (GSE75010 and GSE114691), suggesting that AMIGO2 could be relevant to the pathogenesis of EOPE (Figure 8E).

## Discussion

Our data highlight the disrupted expression of TGF-β superfamily members as a potential etiologic factor in the development of EOPE. We further emphasize the differential expression and putative role of one TGF-β superfamily member, BMP2, in the pathologic condition of EOPE. For the first time, we profile the expression of BMP2 at the fetal-maternal interface in early pregnancy. In addition, our data provide evidence that BMP2 transcriptionally regulates cell adhesion and ECM molecules to promote trophoblast differentiation along the extravillous pathway. Our study also uncovers AMIGO2 as a novel target for BMP2 and sheds new light on the expression and the pro-invasive effects of AMIGO2 in human trophoblasts.

EOPE is associated with shallow EVT invasion and deficiencies in uterine spiral artery remodeling during the first-trimester of pregnancy, and dysregulation of many factors resulting in the trophoblast dysfunction has been implicated in EOPE pathogenesis (Nikuei et al., 2015). Here, we have generated a comprehensive expression profile of TGF-β superfamily-associated genes in the placentas of EOPE versus normotensive women. We found that *TGFB1* was upregulated in EOPE, which supports the elevated expression and serum levels of TGF-β1 in patients with preeclampsia (Djurovic et al., 1997; Peracoli et al., 2008). Blocking TGF-β3 has been shown to restore the invasive capability of EVTs in preeclamptic pregnancies, revealing its suppressive role for EVT invasion (Caniggia et al., 1999). In this context, the downregulation of *TGFB3* in our analysis could suggest some type of compensatory effect with respect to EVT invasion. Supported by our data, activin A (*INHBA*) and inhibin A (*INHA*), but not inhibin B (*INHB*), are increased in patients with preeclampsia (Laivuori et al., 1999; Yair et al., 2001; Silver et al., 2002; Bersinger et al., 2003). Given that TGF-β ligands have been implicated in the control of trophoblast invasion and migration (Graham et al., 1992; Jones et al., 2006; Zhao et al., 2018b), our data support placental contributions to the pathology of EOPE and highlight the TGF-β superfamily as a potential etiologic factor in EOPE. Interestingly, our results have identified that three BMPs subfamily ligand genes (*BMP2*, *BMP4*, *BMP5*) are downregulated in placentas from women with EOPE compared to controls, suggesting that BMP pathways are relevant to the pathogenesis of EOPE. Increased expression of *BMPR2*, which encodes type II receptors for BMPs, could constitute a compensatory mechanism in response to the reduced levels of BMP ligands in EOPE. The dysregulation of BMP pathway components in placental tissues from women with EOPE may provide novel diagnostic and/or therapeutic strategies to manage placenta-related pregnancy disorders.

Previous studies of several mouse models have demonstrated that BMP signaling is involved in the regulation of blastocyst implantation, endometrial stromal cell decidualization, and placental development. BMP2 has been detected in the uterus of mice and its expression is spatiotemporally correlated with embryo implantation during pregnancy (Paria et al., 2001). Female mice with conditional deletion of *Bmp2* in the uterus are infertile due to impaired decidualization of the endometrial stroma (Lee et al., 2007). Moreover, conditional ablation of *Bmpr2* in the uterus causes defective spiral artery remodeling at mid-gestation that results in IUGR and fetal death (Nagashima et al., 2013). In humans, relatively little is known about the expression and roles of BMPs in the uterus and placenta during pregnancy. BMP2 is induced during *in vitro* decidualization of human endometrial stromal cells in response to steroids and cAMP, and addition of BMP2 stimulates decidualization (Li et al., 2007). Additionally, BMP2 has been implicated in regulating human trophoblast invasion and spiral artery remodeling using *in vitro* cell models (Zhao et al., 2018b; Zhao et al., 2020b). Our study provides the first evidence of *BMP2* localization in all trophoblast cell populations of human first-trimester placenta and decidua tissue. Microarray analysis shows an increasing level of *BMP2* in the human placenta during the first trimester of pregnancy. These data suggest that autocrine/paracrine BMP2 produced by both placenta trophoblasts and decidua promotes human trophoblast differentiation along the EVT pathway. Although we did not observe differential expression of BMP2 in GSE75010, analyses from two other datasets (GSE44711, and GSE114691) showed reduced levels of BMP2 in EOPE. The difference across datasets may be due to differences in patients’ ethnicity, platforms used, and study design between different cohorts. Importantly, the RNAscope data support our bioinformatic analysis and reveal that the mRNA expression of BMP2 is abnormal in the EOPE placentas. Our finding of altered expression of BMP2 and receptors in EOPE placentas, combined with the aforementioned mouse work, indicates that BMP2 signaling might be important in human placental development and trophoblast biology. A recent study has shown that the serum concentration of BMP2 is significantly higher in first-trimester pregnancies compared with non-pregnant individuals, suggesting that the developing placenta may be the source of the increased circulating BMP2(You et al., 2021). Future research designed to examine the maternal circulating levels of BMPs in routine blood samples and to compare the BMPs serum levels between EOPE and non-preeclamptic women will provide the theoretical basis for clinical decision-making regarding the use of BMP2 as a potential biomarker for EOPE diagnosis.

Transcriptome analysis has shown BMP2 targets are mainly involved in regulating cell adhesion and ECM organization. The process of trophoblasts invading into the maternal decidua and eventually to the endometrial spiral arteries is accompanied by altered expression of cell adhesion molecules and remodelling of the ECM by various proteinases, including serine proteases, metalloproteinases and collagenases (McEwan et al., 2009). Indeed, we show that BMP2 induces expression of cellular adhesion genes such as *CDH2* and *AMIGO2* as well as ECM-associated genes including ECM components (*COL6A1*, *COL7A1*), ECM receptors (*ITGA2*, *ITGA6*), and proteinases (*ADAM12*, *MMP11*). Combined with our previous studies of BMP2 expression and function in human trophoblast cells, our findings suggest that BMP2 promotes differentiation along the EVT pathway and enhances molecular pathways related to cell adhesion and ECM remodeling.

AMIGO2 is a member of the adhesion molecule with IgG-like domain family of proteins (AMIGO1, 2, 3), which were identified as type I transmembrane proteins induced by the neurite-promoting protein amphoterin in neurons (Kuja-Panula et al., 2003). AMIGO2 is preferentially expressed in the central nervous system and plays an important role in neuronal development and survival (Kuja-Panula et al., 2003; Ono et al., 2003). AMIGO2 has also been linked to the processes of tumorigenicity and metastasis due to its ability to regulate tumour cell adhesion, migration and survival (Rabenau et al., 2004; Fontanals-Cirera et al., 2017; Kanda et al., 2017). In addition, recent studies suggest that AMIGO2 contributes to vascular development and angiogenesis by regulating endothelial cell adhesion, migration, survival, and angiogenesis (Park et al., 2015). Our discovery that AMIGO2 is preferentially expressed in progenitor CTBs and invasive EVTs combined with our finding that disruption of AMIGO2 impairs BMP2-regulated trophoblast cell invasion and endothelial-like tube formation suggest it is required for BMP2-mediated trophoblast invasive and endovascular differentiation. Moreover, the downregulation of placental AMIGO2 in EOPE suggests its involvement in the pathophysiology of EOPE, which is associated with aberrant trophoblast functions. Future studies are required to determine the precise mechanism of how AMIGO2 modulates trophoblast functions. AMIGO2 contains six leucine-rich repeats (LRRs), a single IgG-like domain, a transmembrane domain, and a cytosolic domain (Kuja-Panula et al., 2003). It is possible that AMIGO2 may regulate trophoblast adhesion to ECM and decidua cells via its LRR domains.

Our study combined transcriptomic datasets analyses, *in situ* gene expression profiling, and *in vitro* functional evaluation to elucidate the novel mechanism of BMP2 action in the first-trimester maternal-fetal interface and links the critical role of BMP2 in early trophoblast development to the potential pathological mechanisms for poor placentation in EOPE.

## Data Availability

Data have been deposited in NCBI’s Gene Expression Omnibus repository and are accessible through GEO Series accession number GSE172040.
