# Supplementary material for: Dysregulated BMP2 in the Placenta May Contribute to Early-Onset Preeclampsia by Regulating Human Trophoblast Expression of Extracellular Matrix and Adhesion Molecules
Source: Front Cell Dev Biol. 2021 Dec 14;9:768669. doi: 10.3389/fcell.2021.768669 (PMC8712873; doi:10.3389/fcell.2021.768669)
Supplement: Supplementary file 1 [file Table1.docx]

| **Supplementary Table1 Primers used in study** | | | |
| --- | --- | --- | --- |
| **Name** | **Species** | **Forward 5'to 3'** | **Reverse 5' to 3'** |
| ID1 | Human | CTCTACGACATGAACGGCTGT | TGCTCACCTTGCGGTTCTG |
| ID2 | Human | TCCCAGGGTGTTCTCTTACTTGGACT | GGATCCTTCTGGTATTCACGCTCCA |
| ID3 | Human | CGCGTCATCGACTACATTCT | GAGCTCGGCTGTCTGGAT |
| ID4 | Human | GTGCGATATGAACGACTGCT | CAGGATCTCCACTTTGCTGA |
| ENG | Human | TGCACTTGGCCTACAATTCCA | AGCTGCCCACTCAAGGATCT |
| IL6 | Human | CCTGAACCTTCCAAAGATGGC | TTCACCAGGCAAGTCTCCTCA |
| CAV1 | Human | GCGACCCTAAACACCTCAAC | ATGCCGTCAAAACTGTGTGTC |
| CTGF | Human | GCGTGTGCACCGCCAAAGAT | CAGGGCTGGGCAGACGAACG |
| CCL2 | Human | CAGCCAGATGCAATCAATGCC | TGGAATCCTGAACCCACTTCT |
| AMIGO2 | Human | CCTGGGAACCTTTTCAGACTG | GCAAACGATACTGGAATCCACT |
| CD9 | Human | CCTGCTGTTCGGATTTAACTTCA | TGGTCTGAGAGTCGAATCGGA |
| SPON2 | Human | CGCTGGACCTGTACCCCTA | AGGAGGACGTTATCTCGGTCA |
| SERPINE2 | Human | TGGTGATGAGATACGGCGTAA | GTTAGCCACTGTCACAATGTCTT |
| MMP11 | Human | AAGGTATGGAGCGATGTGACG |    \| GTCCAGGTCTCATCATAGTCGAA \| \| --- \| |
| HMOX1 | Human |    \| AAGACTGCGTTCCTGCTCAAC \| \| --- \| | AAAGCCCTACAGCAACTGTCG |
| COL6A1 | Human | TCAAGAGCCTGCAGTGGATG | TGGACACTTCTTGTCTATGCAG |
| COL7A1 | Human | GTGAGGACTGCCCCTGAG | GACTCCACCTTCGAGACCC |
| FBLN1 | Human | TGCGAATGCAAGACGGGTTA | CCGTAGACGTTGGCACACTC |
| FBLN2 | Human | ACTGTGGGTTCTTACCACTGT | CCACCTGGGAAAATTCTGACTT |
| FBLN5 | Human | CTCACTGTTACCATTCTGGCTC | GACTGGCGATCCAGGTCAAAG |
| HTRA1 | Human | TCCCAACAGTTTGCGCCATAA | CCGGCACCTCTCGTTTAGAAA |
| GAPDH | Human | GAGTCAACGGATTTGGTCGT | GACAAGCTTCCCGTTCTCAG |
