# Supplementary material for: Dysregulated BMP2 in the Placenta May Contribute to Early-Onset Preeclampsia by Regulating Human Trophoblast Expression of Extracellular Matrix and Adhesion Molecules
Source: Front Cell Dev Biol. 2021 Dec 14;9:768669. doi: 10.3389/fcell.2021.768669 (PMC8712873; doi:10.3389/fcell.2021.768669)
Supplement: Supplementary file 2 [file Table2.docx]

| **Supplementary Table 2. Differentially expressed TGFb family genes in placenta (EOPET vs control)** | | | | | | |
| --- | --- | --- | --- | --- | --- | --- |
| PROBEID | Gene Symbol | log2 (Fold Change) | adj.P.Val | Ctrl_mean | EOPET_mean | Significancy (adj.P.Val<0.05 & Fold Change >1.2) |
| 8048639 | INHA | 1.097 | 5.10E-11 | 8.475 | 9.572 | Upregulated |
| 8139207 | INHBA | 0.988 | 1.48E-11 | 11.718 | 12.706 | Upregulated |
| 8164269 | ENG | 0.727 | 3.16E-11 | 11.201 | 11.929 | Upregulated |
| 8008627 | NOG | 0.517 | 5.46E-05 | 7.577 | 8.094 | Upregulated |
| 8037005 | TGFB1 | 0.443 | 4.01E-05 | 9.416 | 9.86 | Upregulated |
| 8092169 | TNFSF10 | 0.403 | 4.01E-05 | 11.057 | 11.46 | Upregulated |
| 8041383 | LTBP1 | 0.382 | 1.91E-07 | 10.392 | 10.774 | Upregulated |
| 8047538 | BMPR2 | 0.277 | 1.70E-08 | 10.837 | 11.115 | Upregulated |
| 8078350 | TGFBR2 | -0.264 | 5.46E-05 | 10.078 | 9.814 | Downregulated |
| 7982597 | THBS1 | -0.294 | 3.23E-02 | 10.35 | 10.056 | Downregulated |
| 8016646 | COL1A1 | -0.301 | 1.28E-03 | 10.217 | 9.916 | Downregulated |
| 7926875 | BAMBI | -0.308 | 4.82E-03 | 8.596 | 8.288 | Downregulated |
| 7979241 | BMP4 | -0.327 | 6.65E-04 | 7.981 | 7.654 | Downregulated |
| 7980316 | TGFB3 | -0.356 | 3.65E-03 | 8.951 | 8.595 | Downregulated |
| 8134263 | COL1A2 | -0.387 | 2.32E-04 | 9.863 | 9.476 | Downregulated |
| 8127193 | BMP5 | -0.436 | 1.46E-05 | 10.108 | 9.672 | Downregulated |
| 7965873 | IGF1 | -0.495 | 9.48E-07 | 8.88 | 8.385 | Downregulated |
| 7909789 | TGFB2 | -0.119 | 2.55E-01 | 8.849 | 8.73 | Not significant |
| 8056005 | ACVR1 | 0.05 | 3.02E-01 | 8.479 | 8.528 | Not significant |
| 8045587 | ACVR2A | -0.011 | 8.44E-01 | 7.982 | 7.971 | Not significant |
| 7955562 | ACVRL1 | -0.254 | 1.67E-03 | 10.512 | 10.258 | Not significant |
| 7955797 | AMHR2 | 0.023 | 5.29E-01 | 6.757 | 6.78 | Not significant |
| 8180337 | ATF4 | 0.078 | 2.90E-02 | 10.746 | 10.825 | Not significant |
| 8145055 | BMP1 | 0.144 | 1.08E-03 | 10.928 | 11.073 | Not significant |
| 8060850 | BMP2 | -0.045 | 5.84E-01 | 7.522 | 7.477 | Not significant |
| 8096070 | BMP3 | 0.062 | 2.02E-01 | 6.014 | 6.076 | Not significant |
| 8116818 | BMP6 | -0.243 | 1.61E-05 | 8.502 | 8.259 | Not significant |
| 8067185 | BMP7 | -0.074 | 3.71E-01 | 8.981 | 8.907 | Not significant |
| 8132250 | BMPER | -0.058 | 2.52E-01 | 7.077 | 7.019 | Not significant |
| 7928855 | BMPR1A | -0.056 | 3.06E-01 | 9.277 | 9.22 | Not significant |
| 7952305 | BMPR1A | -0.031 | 5.84E-01 | 9.046 | 9.015 | Not significant |
| 8096511 | BMPR1B | 0.055 | 8.74E-02 | 5.742 | 5.796 | Not significant |
| 8119088 | CDKN1A | -0.072 | 3.06E-01 | 8.561 | 8.489 | Not significant |
| 7954029 | CDKN1B | 0.097 | 1.77E-02 | 9.687 | 9.784 | Not significant |
| 8160452 | CDKN2B | 0.021 | 5.54E-01 | 7.692 | 7.713 | Not significant |
| 8084496 | CHRD | 0.064 | 3.42E-02 | 7.05 | 7.113 | Not significant |
| 7965410 | DCN | -0.04 | 5.84E-01 | 11.386 | 11.346 | Not significant |
| 8056784 | DLX2 | 0.061 | 1.20E-01 | 6.687 | 6.748 | Not significant |
| 7954090 | EMP1 | -0.178 | 1.61E-02 | 9.427 | 9.249 | Not significant |
| 7975779 | FOS | 0.137 | 3.84E-01 | 10.095 | 10.232 | Not significant |
| 8105302 | FST | -0.062 | 5.05E-01 | 8.019 | 7.957 | Not significant |
| 8024485 | GADD45B | 0.027 | 5.84E-01 | 8.494 | 8.521 | Not significant |
| 7933366 | GDF2 | 0.045 | 2.52E-01 | 7.324 | 7.37 | Not significant |
| 7960828 | GDF3 | 0.069 | 4.72E-02 | 4.574 | 4.643 | Not significant |
| 8065905 | GDF5 | 0.098 | 5.88E-03 | 5.873 | 5.971 | Not significant |
| 8151906 | GDF6 | 0.027 | 4.64E-01 | 6.556 | 6.583 | Not significant |
| 8040479 | GDF7 | 0.087 | 1.28E-03 | 7.644 | 7.731 | Not significant |
| 7981106 | GSC | 0.057 | 8.69E-02 | 7.39 | 7.447 | Not significant |
| 7995895 | HERPUD1 | 0.224 | 2.32E-04 | 11.019 | 11.243 | Not significant |
| 8143307 | HIPK2 | -0.072 | 1.27E-01 | 9.01 | 8.938 | Not significant |
| 8061564 | ID1 | 0.137 | 9.00E-02 | 9.362 | 9.499 | Not significant |
| 8040103 | ID2 | -0.149 | 2.23E-02 | 9.177 | 9.028 | Not significant |
| 8135514 | IFRD1 | -0.16 | 2.17E-03 | 8.834 | 8.675 | Not significant |
| 8139488 | IGFBP3 | 0.167 | 2.90E-02 | 10.396 | 10.562 | Not significant |
| 8131803 | IL6 | -0.048 | 3.46E-01 | 6.322 | 6.274 | Not significant |
| 8044927 | INHBB | 0.088 | 4.28E-02 | 7.733 | 7.821 | Not significant |
| 7916609 | JUN | -0.012 | 8.90E-01 | 8 | 7.987 | Not significant |
| 8026047 | JUNB | 0.26 | 4.87E-06 | 9.979 | 10.24 | Not significant |
| 7924663 | LEFTY1 | 0.042 | 2.61E-01 | 7.535 | 7.577 | Not significant |
| 7980152 | LTBP2 | -0.066 | 3.14E-01 | 8.379 | 8.313 | Not significant |
| 8091972 | MECOM | 0.142 | 1.46E-02 | 7.72 | 7.862 | Not significant |
| 8148317 | MYC | -0.196 | 1.08E-03 | 8.71 | 8.514 | Not significant |
| 7934156 | NODAL | 0.059 | 1.35E-01 | 5.79 | 5.849 | Not significant |
| 8076195 | PDGFB | -0.003 | 9.21E-01 | 7.873 | 7.87 | Not significant |
| 7928429 | PLAU | 0.089 | 3.64E-01 | 9.79 | 9.879 | Not significant |
| 8070194 | RUNX1 | -0.03 | 4.45E-01 | 8.163 | 8.133 | Not significant |
| 8135069 | SERPINE1 | 0.178 | 1.85E-02 | 11.724 | 11.902 | Not significant |
| 8097657 | SMAD1 | -0.127 | 4.44E-02 | 7.88 | 7.753 | Not significant |
| 8023191 | SMAD2 | 0.042 | 3.68E-01 | 8.709 | 8.751 | Not significant |
| 7984364 | SMAD3 | -0.032 | 5.53E-01 | 8.004 | 7.971 | Not significant |
| 8021228 | SMAD4 | 0.098 | 5.88E-03 | 10.79 | 10.888 | Not significant |
| 8108238 | SMAD5 | -0.133 | 1.63E-02 | 8.898 | 8.766 | Not significant |
| 8023220 | SMAD7 | -0.092 | 8.32E-02 | 8.344 | 8.251 | Not significant |
| 7971015 | SMAD9 | 0.138 | 5.75E-03 | 7.642 | 7.78 | Not significant |
| 8141241 | SMURF1 | 0.058 | 1.49E-01 | 9.462 | 9.52 | Not significant |
| 8117165 | SOX4 | 0.047 | 1.54E-01 | 7.726 | 7.773 | Not significant |
| 8057744 | STAT1 | 0.033 | 6.91E-01 | 9.597 | 9.63 | Not significant |
| 7995206 | TGFB1I1 | -0.127 | 8.32E-04 | 8.765 | 8.638 | Not significant |
| 8108217 | TGFBI | -0.118 | 3.18E-01 | 9.869 | 9.751 | Not significant |
| 8156826 | TGFBR1 | 0.052 | 2.47E-01 | 11.097 | 11.149 | Not significant |
| 7917649 | TGFBR3 | -0.11 | 1.85E-02 | 11.4 | 11.29 | Not significant |
| 8054364 | TGFBRAP1 | -0.035 | 5.16E-01 | 8.544 | 8.509 | Not significant |
| 8180319 | TGIF1 | 0.037 | 3.71E-01 | 9.878 | 9.915 | Not significant |
| 7971350 | TSC22D1 | 0.247 | 4.84E-07 | 8.426 | 8.673 | Not significant |
