# Supplementary material for: Dysregulated BMP2 in the Placenta May Contribute to Early-Onset Preeclampsia by Regulating Human Trophoblast Expression of Extracellular Matrix and Adhesion Molecules
Source: Front Cell Dev Biol. 2021 Dec 14;9:768669. doi: 10.3389/fcell.2021.768669 (PMC8712873; doi:10.3389/fcell.2021.768669)
Supplement: Supplementary file 3 [file Table3.docx]

| **Supplementary Table 3. Differentially expressed genes in primary trophoblasts (BMP2 vs Control)** | | | | | | | |
| --- | --- | --- | --- | --- | --- | --- | --- |
| gene | Ctrl | BMP2 | log2 (fold_change) | test_stat | p_value | q_value | significant |
| ACTC1 | 0.15891 | 5.98932 | 5.23615 | 5.45087 | 5.00E-05 | 2.76E-03 | yes |
| LOC100130417 | 0.10868 | 2.59115 | 4.57543 | 2.84357 | 5.00E-05 | 2.76E-03 | yes |
| SYT15 | 0.14446 | 1.67197 | 3.53282 | 2.62378 | 5.00E-05 | 2.76E-03 | yes |
| ELN | 0.53034 | 6.03694 | 3.50882 | 4.50305 | 5.00E-05 | 2.76E-03 | yes |
| SMAD6 | 0.99189 | 10.51670 | 3.40635 | 5.27660 | 5.00E-05 | 2.76E-03 | yes |
| ID3 | 24.76530 | 213.17200 | 3.10563 | 8.38375 | 5.00E-05 | 2.76E-03 | yes |
| COMP | 0.07866 | 0.66836 | 3.08695 | 2.28955 | 1.50E-04 | 7.06E-03 | yes |
| ID4 | 0.84350 | 7.00999 | 3.05496 | 4.80627 | 5.00E-05 | 2.76E-03 | yes |
| CHRNA9 | 0.09551 | 0.77765 | 3.02542 | 2.11851 | 1.60E-03 | 4.84E-02 | yes |
| ID1 | 25.93270 | 194.17200 | 2.90449 | 7.34077 | 5.00E-05 | 2.76E-03 | yes |
| NOG | 2.25422 | 16.13460 | 2.83946 | 4.70186 | 5.00E-05 | 2.76E-03 | yes |
| DLX2 | 0.36735 | 2.62382 | 2.83645 | 3.03482 | 5.00E-05 | 2.76E-03 | yes |
| SMAD9 | 0.99802 | 6.90723 | 2.79096 | 5.16356 | 5.00E-05 | 2.76E-03 | yes |
| ID2 | 17.74290 | 111.23800 | 2.64834 | 6.96165 | 5.00E-05 | 2.76E-03 | yes |
| SGCG | 0.24637 | 1.49999 | 2.60606 | 1.98321 | 9.50E-04 | 3.18E-02 | yes |
| DUSP2 | 0.31139 | 1.82998 | 2.55504 | 2.26901 | 5.00E-05 | 2.76E-03 | yes |
| LINC00890 | 0.12974 | 0.73921 | 2.51039 | 2.37465 | 5.00E-05 | 2.76E-03 | yes |
| EGLN3 | 0.47303 | 2.56974 | 2.44162 | 2.76889 | 5.00E-05 | 2.76E-03 | yes |
| C9orf135 | 0.20533 | 1.07188 | 2.38413 | 1.15857 | 2.00E-04 | 8.81E-03 | yes |
| PGF | 0.62834 | 3.14483 | 2.32336 | 2.55225 | 5.00E-05 | 2.76E-03 | yes |
| BMF | 0.99136 | 4.74348 | 2.25846 | 3.82154 | 5.00E-05 | 2.76E-03 | yes |
| ATOH8 | 2.17636 | 10.08700 | 2.21251 | 5.11705 | 5.00E-05 | 2.76E-03 | yes |
| PREX2 | 0.15865 | 0.67921 | 2.09806 | 2.13117 | 5.00E-05 | 2.76E-03 | yes |
| CHRD | 0.33904 | 1.39329 | 2.03897 | 2.20462 | 5.00E-05 | 2.76E-03 | yes |
| PLCXD3 | 1.17284 | 4.74916 | 2.01767 | 4.24401 | 5.00E-05 | 2.76E-03 | yes |
| TSPAN11 | 0.44121 | 1.77407 | 2.00753 | 2.75977 | 5.00E-05 | 2.76E-03 | yes |
| HEY1 | 0.45369 | 1.77389 | 1.96713 | 2.07456 | 5.00E-05 | 2.76E-03 | yes |
| TNFAIP8L3 | 0.20693 | 0.78991 | 1.93253 | 1.65447 | 1.45E-03 | 4.50E-02 | yes |
| GPC4 | 1.91161 | 6.93452 | 1.85901 | 3.55690 | 5.00E-05 | 2.76E-03 | yes |
| SMAD7 | 8.25222 | 29.91130 | 1.85784 | 4.86369 | 5.00E-05 | 2.76E-03 | yes |
| IGFBP3 | 29.15710 | 104.98100 | 1.84822 | 5.12459 | 5.00E-05 | 2.76E-03 | yes |
| TSPAN8 | 1.10896 | 3.93538 | 1.82729 | 1.95875 | 2.00E-04 | 8.81E-03 | yes |
| LUZP2 | 0.24681 | 0.86252 | 1.80516 | 1.96904 | 5.00E-05 | 2.76E-03 | yes |
| BAMBI | 14.68800 | 51.04580 | 1.79715 | 4.74464 | 5.00E-05 | 2.76E-03 | yes |
| CCDC85A | 1.95333 | 6.61331 | 1.75943 | 3.33783 | 5.00E-05 | 2.76E-03 | yes |
| ADGRB3 | 0.22157 | 0.74346 | 1.74647 | 1.97710 | 5.00E-05 | 2.76E-03 | yes |
| DSP | 15.83330 | 52.38990 | 1.72633 | 5.22036 | 5.00E-05 | 2.76E-03 | yes |
| RBM24 | 11.78370 | 36.96110 | 1.64921 | 4.51195 | 5.00E-05 | 2.76E-03 | yes |
| FGFR3 | 0.53186 | 1.64139 | 1.62580 | 2.20215 | 5.00E-05 | 2.76E-03 | yes |
| UNC5B | 1.92701 | 5.90748 | 1.61618 | 3.64640 | 5.00E-05 | 2.76E-03 | yes |
| EFHD1 | 6.83609 | 20.57770 | 1.58984 | 3.52206 | 5.00E-05 | 2.76E-03 | yes |
| COL9A2 | 0.26517 | 0.79581 | 1.58549 | 1.55718 | 8.50E-04 | 2.94E-02 | yes |
| VASH2 | 0.51944 | 1.55532 | 1.58217 | 2.11601 | 1.00E-04 | 5.02E-03 | yes |
| MEX3A | 1.74154 | 5.11261 | 1.55369 | 3.38344 | 5.00E-05 | 2.76E-03 | yes |
| CCDC68 | 2.63164 | 7.64053 | 1.53771 | 3.33006 | 5.00E-05 | 2.76E-03 | yes |
| HMCN1 | 1.72662 | 4.98202 | 1.52878 | 2.24043 | 5.00E-05 | 2.76E-03 | yes |
| PLCL1 | 0.31491 | 0.87731 | 1.47813 | 1.88408 | 1.50E-04 | 7.06E-03 | yes |
| SOX4 | 33.04300 | 91.68350 | 1.47232 | 4.58945 | 5.00E-05 | 2.76E-03 | yes |
| ITGA6 | 35.19110 | 95.56420 | 1.44126 | 4.16005 | 5.00E-05 | 2.76E-03 | yes |
| SALL1 | 3.84134 | 10.17640 | 1.40555 | 3.53183 | 5.00E-05 | 2.76E-03 | yes |
| CD24 | 6.59245 | 16.70110 | 1.34106 | 3.01407 | 5.00E-05 | 2.76E-03 | yes |
| NDRG4 | 3.88310 | 9.79711 | 1.33515 | 2.82782 | 5.00E-05 | 2.76E-03 | yes |
| PCDH10 | 9.74658 | 24.51640 | 1.33078 | 3.66646 | 5.00E-05 | 2.76E-03 | yes |
| INHBA | 35.26640 | 88.24920 | 1.32329 | 4.07211 | 5.00E-05 | 2.76E-03 | yes |
| GPR137C | 0.60124 | 1.49347 | 1.31267 | 1.70010 | 6.00E-04 | 2.21E-02 | yes |
| SLC8A1 | 3.42202 | 8.49186 | 1.31123 | 3.37412 | 5.00E-05 | 2.76E-03 | yes |
| CRYAB | 13.85820 | 34.12380 | 1.30004 | 2.64789 | 5.00E-05 | 2.76E-03 | yes |
| FAM196B | 3.97629 | 9.68782 | 1.28475 | 2.87890 | 5.00E-05 | 2.76E-03 | yes |
| LYPD6B | 2.38740 | 5.71424 | 1.25912 | 1.68526 | 8.50E-04 | 2.94E-02 | yes |
| DGKI | 2.59829 | 6.18732 | 1.25175 | 2.74954 | 5.00E-05 | 2.76E-03 | yes |
| LINC01503 | 5.49050 | 13.02050 | 1.24577 | 1.77451 | 2.00E-04 | 8.81E-03 | yes |
| LOC728392 | 12.09690 | 28.07850 | 1.21484 | 2.93456 | 5.00E-05 | 2.76E-03 | yes |
| ADRA2A | 6.73834 | 15.61170 | 1.21217 | 2.97025 | 5.00E-05 | 2.76E-03 | yes |
| IFITM10 | 2.08840 | 4.78983 | 1.19758 | 2.28576 | 5.00E-05 | 2.76E-03 | yes |
| MEGF9 | 3.12279 | 7.08888 | 1.18272 | 2.95164 | 5.00E-05 | 2.76E-03 | yes |
| PARM1 | 2.64705 | 5.94728 | 1.16785 | 2.59874 | 5.00E-05 | 2.76E-03 | yes |
| HTRA1 | 267.64600 | 598.60400 | 1.16127 | 3.24913 | 5.00E-05 | 2.76E-03 | yes |
| DACT1 | 2.18036 | 4.87581 | 1.16108 | 2.21247 | 5.00E-05 | 2.76E-03 | yes |
| PCDH19 | 0.52589 | 1.17364 | 1.15816 | 1.88267 | 1.00E-04 | 5.02E-03 | yes |
| SEMA3D | 0.88954 | 1.97856 | 1.15332 | 1.96133 | 1.00E-04 | 5.02E-03 | yes |
| NOX4 | 0.73946 | 1.64342 | 1.15217 | 1.33570 | 6.50E-04 | 2.35E-02 | yes |
| GRIK2 | 5.53133 | 12.22610 | 1.14426 | 3.12755 | 5.00E-05 | 2.76E-03 | yes |
| STMN2 | 6.75112 | 14.88790 | 1.14094 | 2.17995 | 5.00E-05 | 2.76E-03 | yes |
| SYTL5 | 26.23050 | 56.11340 | 1.09710 | 3.49601 | 5.00E-05 | 2.76E-03 | yes |
| SCUBE3 | 12.42100 | 26.50160 | 1.09330 | 3.22076 | 5.00E-05 | 2.76E-03 | yes |
| GPR137B | 20.00180 | 42.14440 | 1.07521 | 3.12182 | 5.00E-05 | 2.76E-03 | yes |
| CACNA1G | 0.41553 | 0.87365 | 1.07210 | 1.44673 | 5.00E-05 | 2.76E-03 | yes |
| ZNF804A | 3.02333 | 6.32738 | 1.06547 | 2.44669 | 5.00E-05 | 2.76E-03 | yes |
| SERPINE2 | 323.72800 | 675.42200 | 1.06101 | 2.57365 | 5.00E-05 | 2.76E-03 | yes |
| PABPC4L | 0.59150 | 1.23241 | 1.05903 | 1.43486 | 1.20E-03 | 3.89E-02 | yes |
| SGK223 | 6.51252 | 13.51770 | 1.05356 | 2.87082 | 5.00E-05 | 2.76E-03 | yes |
| HIP1 | 7.47394 | 15.45790 | 1.04840 | 3.29462 | 5.00E-05 | 2.76E-03 | yes |
| MAFB | 1.27044 | 2.62395 | 1.04642 | 1.60920 | 1.50E-04 | 7.06E-03 | yes |
| EDN1 | 2.47232 | 5.07656 | 1.03798 | 1.64061 | 6.00E-04 | 2.21E-02 | yes |
| ZSWIM5 | 2.14594 | 4.39583 | 1.03452 | 2.26030 | 5.00E-05 | 2.76E-03 | yes |
| SLC7A8 | 4.99450 | 10.21770 | 1.03265 | 2.43915 | 5.00E-05 | 2.76E-03 | yes |
| NPR3 | 0.45307 | 0.92505 | 1.02981 | 1.42381 | 1.55E-03 | 4.75E-02 | yes |
| SHISA9 | 1.80927 | 3.68189 | 1.02504 | 1.89689 | 5.00E-05 | 2.76E-03 | yes |
| SAT1 | 69.15880 | 139.67100 | 1.01405 | 3.03368 | 5.00E-05 | 2.76E-03 | yes |
| S1PR1 | 6.59645 | 13.28870 | 1.01044 | 2.50367 | 5.00E-05 | 2.76E-03 | yes |
| ZNF521 | 2.18201 | 4.38203 | 1.00594 | 2.11461 | 5.00E-05 | 2.76E-03 | yes |
| HIP1R | 8.60370 | 17.21380 | 1.00053 | 2.81851 | 5.00E-05 | 2.76E-03 | yes |
| LGALS9 | 6.77735 | 13.50210 | 0.99439 | 2.07479 | 1.00E-04 | 5.02E-03 | yes |
| CSF2RB | 1.64449 | 3.26990 | 0.99161 | 1.86452 | 5.00E-05 | 2.76E-03 | yes |
| ANKRD1 | 18.66330 | 36.74540 | 0.97736 | 2.60977 | 5.00E-05 | 2.76E-03 | yes |
| MARCKSL1 | 74.35470 | 146.38800 | 0.97730 | 3.16556 | 5.00E-05 | 2.76E-03 | yes |
| COL24A1 | 1.35585 | 2.64185 | 0.96235 | 1.89109 | 5.00E-05 | 2.76E-03 | yes |
| LIMK2 | 18.70820 | 36.41970 | 0.96105 | 2.95983 | 5.00E-05 | 2.76E-03 | yes |
| IRS1 | 11.25360 | 21.48090 | 0.93267 | 3.00869 | 5.00E-05 | 2.76E-03 | yes |
| MEX3B | 3.01908 | 5.76144 | 0.93232 | 1.90827 | 5.00E-05 | 2.76E-03 | yes |
| TUBB3 | 85.92790 | 163.83800 | 0.93107 | 2.94550 | 5.00E-05 | 2.76E-03 | yes |
| SPON2 | 532.63600 | 1013.74000 | 0.92847 | 2.36028 | 5.00E-05 | 2.76E-03 | yes |
| C8orf4 | 13.03000 | 24.75130 | 0.92566 | 2.39701 | 5.00E-05 | 2.76E-03 | yes |
| GPRC5C | 3.17432 | 6.02079 | 0.92350 | 1.53756 | 9.50E-04 | 3.18E-02 | yes |
| CADM1 | 12.16870 | 23.06990 | 0.92283 | 2.82132 | 5.00E-05 | 2.76E-03 | yes |
| LRP1B | 0.55445 | 1.04925 | 0.92022 | 1.80708 | 3.50E-04 | 1.42E-02 | yes |
| MEGF6 | 1.31213 | 2.46215 | 0.90801 | 1.76944 | 1.00E-04 | 5.02E-03 | yes |
| RAB31 | 23.33970 | 43.73370 | 0.90596 | 2.86265 | 5.00E-05 | 2.76E-03 | yes |
| PRR5L | 6.40484 | 11.98040 | 0.90344 | 2.37000 | 5.00E-05 | 2.76E-03 | yes |
| PIK3R1 | 11.40750 | 21.31110 | 0.90163 | 2.71787 | 5.00E-05 | 2.76E-03 | yes |
| PRSS35 | 2.21967 | 4.14422 | 0.90075 | 1.50234 | 1.20E-03 | 3.89E-02 | yes |
| IGFN1 | 0.59038 | 1.09128 | 0.88632 | 1.54852 | 7.50E-04 | 2.65E-02 | yes |
| LMO7 | 34.09540 | 62.49910 | 0.87426 | 2.59742 | 5.00E-05 | 2.76E-03 | yes |
| FAM26E | 4.25246 | 7.77194 | 0.86998 | 1.98883 | 5.00E-05 | 2.76E-03 | yes |
| NACC2 | 13.08270 | 23.81730 | 0.86435 | 2.77734 | 5.00E-05 | 2.76E-03 | yes |
| CDH2 | 39.70540 | 71.90190 | 0.85669 | 2.73248 | 5.00E-05 | 2.76E-03 | yes |
| KRT7 | 6.49156 | 11.72610 | 0.85308 | 1.63223 | 1.50E-04 | 7.06E-03 | yes |
| OSGIN2 | 9.09336 | 16.39760 | 0.85060 | 2.46936 | 5.00E-05 | 2.76E-03 | yes |
| SLC6A6 | 19.47340 | 35.09940 | 0.84994 | 2.52835 | 5.00E-05 | 2.76E-03 | yes |
| KCNH1 | 2.23180 | 4.00586 | 0.84391 | 2.03578 | 5.00E-05 | 2.76E-03 | yes |
| KANK1 | 16.06310 | 28.82280 | 0.84346 | 2.63012 | 5.00E-05 | 2.76E-03 | yes |
| SLC25A15 | 3.71349 | 6.65494 | 0.84165 | 1.88462 | 5.00E-05 | 2.76E-03 | yes |
| FBLN2 | 11.83260 | 21.07410 | 0.83270 | 2.17010 | 5.00E-05 | 2.76E-03 | yes |
| BIRC2 | 31.78800 | 56.44180 | 0.82828 | 2.56813 | 5.00E-05 | 2.76E-03 | yes |
| GALNT18 | 7.26243 | 12.78260 | 0.81566 | 1.88870 | 5.00E-05 | 2.76E-03 | yes |
| WWC2 | 13.66960 | 24.01470 | 0.81295 | 2.60136 | 5.00E-05 | 2.76E-03 | yes |
| HMOX1 | 50.38050 | 88.42530 | 0.81159 | 2.42347 | 5.00E-05 | 2.76E-03 | yes |
| SGK1 | 42.66710 | 74.82920 | 0.81048 | 2.50645 | 5.00E-05 | 2.76E-03 | yes |
| DUSP10 | 17.42720 | 30.51290 | 0.80808 | 2.34569 | 5.00E-05 | 2.76E-03 | yes |
| SCG2 | 5.14560 | 8.98437 | 0.80408 | 1.65908 | 3.50E-04 | 1.42E-02 | yes |
| AMD1 | 41.61900 | 72.30810 | 0.79692 | 2.15414 | 5.00E-05 | 2.76E-03 | yes |
| MRAS | 20.68330 | 35.85430 | 0.79368 | 2.49531 | 5.00E-05 | 2.76E-03 | yes |
| FGFRL1 | 13.86120 | 23.97730 | 0.79062 | 2.34315 | 5.00E-05 | 2.76E-03 | yes |
| LFNG | 4.13331 | 7.14257 | 0.78915 | 1.49950 | 1.10E-03 | 3.61E-02 | yes |
| LOC90246 | 3.51356 | 6.06760 | 0.78819 | 1.53767 | 1.15E-03 | 3.76E-02 | yes |
| FOXO1 | 4.45645 | 7.65663 | 0.78081 | 2.01444 | 5.00E-05 | 2.76E-03 | yes |
| ENG | 150.79900 | 258.07600 | 0.77517 | 2.29174 | 5.00E-05 | 2.76E-03 | yes |
| JADE3 | 2.50295 | 4.28053 | 0.77416 | 1.64191 | 2.50E-04 | 1.07E-02 | yes |
| ADAMTS19 | 3.12764 | 5.33843 | 0.77134 | 1.77244 | 1.00E-04 | 5.02E-03 | yes |
| ENAH | 9.97234 | 16.99510 | 0.76912 | 2.50637 | 5.00E-05 | 2.76E-03 | yes |
| MARCKS | 97.98050 | 166.72200 | 0.76688 | 2.30306 | 5.00E-05 | 2.76E-03 | yes |
| YOD1 | 2.24666 | 3.81066 | 0.76226 | 1.71743 | 3.00E-04 | 1.25E-02 | yes |
| MYO6 | 14.50070 | 24.58160 | 0.76145 | 2.44189 | 5.00E-05 | 2.76E-03 | yes |
| TPM1 | 657.55300 | 1112.62000 | 0.75879 | 1.86367 | 5.00E-05 | 2.76E-03 | yes |
| CCNG2 | 11.77730 | 19.91020 | 0.75749 | 2.35714 | 5.00E-05 | 2.76E-03 | yes |
| ZBTB18 | 2.45953 | 4.14222 | 0.75202 | 1.46519 | 1.55E-03 | 4.75E-02 | yes |
| PFKFB2 | 3.02158 | 5.05100 | 0.74127 | 1.67495 | 2.50E-04 | 1.07E-02 | yes |
| FBXO32 | 5.32385 | 8.88855 | 0.73948 | 1.74303 | 1.50E-04 | 7.06E-03 | yes |
| ADAMTS2 | 9.03895 | 15.08450 | 0.73884 | 1.63530 | 1.50E-04 | 7.06E-03 | yes |
| MKX | 3.09480 | 5.15847 | 0.73710 | 1.52942 | 5.50E-04 | 2.07E-02 | yes |
| NUAK1 | 12.23000 | 20.30900 | 0.73169 | 2.33072 | 5.00E-05 | 2.76E-03 | yes |
| NPC1 | 20.28800 | 33.64590 | 0.72980 | 2.17343 | 5.00E-05 | 2.76E-03 | yes |
| TEAD2 | 17.39040 | 28.82630 | 0.72909 | 2.07762 | 5.00E-05 | 2.76E-03 | yes |
| NYNRIN | 2.04591 | 3.36894 | 0.71955 | 1.65183 | 2.00E-04 | 8.81E-03 | yes |
| MAGI1 | 2.23963 | 3.68118 | 0.71690 | 1.66115 | 4.50E-04 | 1.75E-02 | yes |
| GABARAPL1 | 151.48900 | 248.66400 | 0.71499 | 2.17523 | 5.00E-05 | 2.76E-03 | yes |
| FBLN5 | 195.47400 | 320.35200 | 0.71268 | 2.07427 | 5.00E-05 | 2.76E-03 | yes |
| LPCAT2 | 18.60900 | 30.37930 | 0.70708 | 2.24303 | 5.00E-05 | 2.76E-03 | yes |
| ACTA2 | 179.51200 | 291.24900 | 0.69818 | 1.76834 | 2.50E-04 | 1.07E-02 | yes |
| SKIL | 11.67200 | 18.93060 | 0.69766 | 2.23035 | 5.00E-05 | 2.76E-03 | yes |
| ANKH | 4.30990 | 6.97363 | 0.69426 | 1.94611 | 5.00E-05 | 2.76E-03 | yes |
| ANGPTL2 | 21.44120 | 34.64010 | 0.69206 | 2.14549 | 5.00E-05 | 2.76E-03 | yes |
| SEMA3C | 21.61820 | 34.91350 | 0.69154 | 2.08603 | 5.00E-05 | 2.76E-03 | yes |
| KCNE4 | 4.32723 | 6.98356 | 0.69052 | 1.49803 | 9.00E-04 | 3.09E-02 | yes |
| ADGRL2 | 8.09677 | 13.02070 | 0.68539 | 2.02248 | 5.00E-05 | 2.76E-03 | yes |
| PLEKHO1 | 25.33020 | 40.70470 | 0.68434 | 2.07328 | 5.00E-05 | 2.76E-03 | yes |
| MCAM | 10.75490 | 17.25540 | 0.68206 | 1.83070 | 1.00E-04 | 5.02E-03 | yes |
| FZD1 | 6.09852 | 9.76849 | 0.67968 | 1.81410 | 5.00E-05 | 2.76E-03 | yes |
| TBX2 | 10.77780 | 17.25880 | 0.67927 | 1.93168 | 5.00E-05 | 2.76E-03 | yes |
| CTGF | 199.56800 | 318.46200 | 0.67424 | 1.86213 | 5.00E-05 | 2.76E-03 | yes |
| MMP11 | 50.12420 | 79.96030 | 0.67378 | 2.05261 | 5.00E-05 | 2.76E-03 | yes |
| AGAP3 | 31.54900 | 50.01080 | 0.66465 | 2.07109 | 5.00E-05 | 2.76E-03 | yes |
| GLI2 | 3.41217 | 5.40158 | 0.66269 | 1.71812 | 5.00E-05 | 2.76E-03 | yes |
| MUM1L1 | 4.23475 | 6.66492 | 0.65431 | 1.52095 | 9.50E-04 | 3.18E-02 | yes |
| CROT | 29.34490 | 46.12890 | 0.65256 | 1.88228 | 5.00E-05 | 2.76E-03 | yes |
| DUSP1 | 30.54460 | 47.87060 | 0.64822 | 1.95990 | 5.00E-05 | 2.76E-03 | yes |
| PGM2L1 | 2.44848 | 3.82467 | 0.64345 | 1.61715 | 3.50E-04 | 1.42E-02 | yes |
| CAMK1G | 8.01402 | 12.49030 | 0.64021 | 1.55395 | 6.00E-04 | 2.21E-02 | yes |
| COL13A1 | 10.81140 | 16.83680 | 0.63907 | 1.71181 | 2.00E-04 | 8.81E-03 | yes |
| SSBP3 | 9.22641 | 14.36350 | 0.63857 | 1.74343 | 1.50E-04 | 7.06E-03 | yes |
| RUNX1 | 7.10791 | 11.04830 | 0.63633 | 1.71662 | 5.00E-05 | 2.76E-03 | yes |
| SMCHD1 | 8.40757 | 13.03570 | 0.63271 | 1.97431 | 5.00E-05 | 2.76E-03 | yes |
| TP53I3 | 44.85030 | 69.35680 | 0.62892 | 1.83927 | 5.00E-05 | 2.76E-03 | yes |
| KCTD15 | 6.92765 | 10.70250 | 0.62751 | 1.68008 | 2.50E-04 | 1.07E-02 | yes |
| MYO10 | 14.60840 | 22.52390 | 0.62465 | 1.98323 | 5.00E-05 | 2.76E-03 | yes |
| STK38L | 6.12310 | 9.41767 | 0.62111 | 1.71192 | 2.00E-04 | 8.81E-03 | yes |
| IER5L | 20.81630 | 31.95790 | 0.61846 | 1.90434 | 5.00E-05 | 2.76E-03 | yes |
| ITGA2 | 27.99870 | 42.80550 | 0.61244 | 1.94291 | 5.00E-05 | 2.76E-03 | yes |
| SLC44A1 | 10.98780 | 16.78160 | 0.61098 | 1.97916 | 5.00E-05 | 2.76E-03 | yes |
| PLXNB3 | 4.66617 | 7.12247 | 0.61014 | 1.66681 | 3.00E-04 | 1.25E-02 | yes |
| NCAM1 | 6.73088 | 10.26900 | 0.60943 | 1.71902 | 5.00E-05 | 2.76E-03 | yes |
| SNX25 | 10.65950 | 16.20940 | 0.60470 | 1.70858 | 2.00E-04 | 8.81E-03 | yes |
| DIO2 | 6.60985 | 10.03560 | 0.60243 | 1.67601 | 1.50E-04 | 7.06E-03 | yes |
| ATXN1 | 4.20167 | 6.36819 | 0.59992 | 1.80148 | 5.00E-05 | 2.76E-03 | yes |
| KLHL24 | 5.70474 | 8.61072 | 0.59397 | 1.74208 | 5.00E-05 | 2.76E-03 | yes |
| IL13RA1 | 33.50780 | 50.37780 | 0.58829 | 1.92103 | 5.00E-05 | 2.76E-03 | yes |
| F2RL1 | 36.40970 | 54.73930 | 0.58826 | 1.87045 | 5.00E-05 | 2.76E-03 | yes |
| SH3KBP1 | 23.35770 | 34.99320 | 0.58318 | 1.85417 | 1.00E-04 | 5.02E-03 | yes |
| NOL4L | 3.94264 | 5.90075 | 0.58174 | 1.51908 | 7.00E-04 | 2.48E-02 | yes |
| MAPK8 | 11.17090 | 16.66480 | 0.57706 | 1.77440 | 5.00E-05 | 2.76E-03 | yes |
| ADAM12 | 10.08830 | 15.04170 | 0.57628 | 1.59942 | 1.50E-04 | 7.06E-03 | yes |
| KIF3C | 8.58923 | 12.77070 | 0.57223 | 1.72197 | 1.50E-04 | 7.06E-03 | yes |
| NCS1 | 22.94600 | 34.08960 | 0.57109 | 1.84510 | 5.00E-05 | 2.76E-03 | yes |
| SNX30 | 4.31263 | 6.39472 | 0.56831 | 1.60059 | 2.00E-04 | 8.81E-03 | yes |
| GALNT10 | 15.15600 | 22.40150 | 0.56371 | 1.79531 | 5.00E-05 | 2.76E-03 | yes |
| IVNS1ABP | 17.69820 | 26.13580 | 0.56242 | 1.79214 | 5.00E-05 | 2.76E-03 | yes |
| FRY | 5.74558 | 8.43956 | 0.55471 | 1.66055 | 2.00E-04 | 8.81E-03 | yes |
| GADD45B | 32.47840 | 47.66400 | 0.55342 | 1.60654 | 2.00E-04 | 8.81E-03 | yes |
| TNC | 19.14060 | 28.03100 | 0.55039 | 1.66025 | 3.00E-04 | 1.25E-02 | yes |
| NEGR1 | 6.14003 | 8.97163 | 0.54713 | 1.54978 | 6.50E-04 | 2.35E-02 | yes |
| SYNE1 | 2.07092 | 3.02553 | 0.54692 | 1.68393 | 2.00E-04 | 8.81E-03 | yes |
| SPECC1 | 19.17640 | 28.00610 | 0.54641 | 1.72732 | 1.50E-04 | 7.06E-03 | yes |
| UST | 6.64417 | 9.69070 | 0.54451 | 1.43581 | 1.40E-03 | 4.38E-02 | yes |
| ZCCHC14 | 8.01766 | 11.68140 | 0.54296 | 1.67058 | 1.00E-04 | 5.02E-03 | yes |
| SPRY2 | 16.71610 | 24.32890 | 0.54143 | 1.50452 | 7.00E-04 | 2.48E-02 | yes |
| DDR1 | 24.36530 | 35.43910 | 0.54052 | 1.65757 | 3.50E-04 | 1.42E-02 | yes |
| KCMF1 | 8.88379 | 12.88080 | 0.53598 | 1.68513 | 5.00E-05 | 2.76E-03 | yes |
| KAZN | 10.46350 | 15.15390 | 0.53432 | 1.52273 | 6.50E-04 | 2.35E-02 | yes |
| HSPG2 | 23.15870 | 33.52530 | 0.53370 | 1.64844 | 3.50E-04 | 1.42E-02 | yes |
| FOXN3 | 8.65633 | 12.51910 | 0.53230 | 1.65722 | 2.00E-04 | 8.81E-03 | yes |
| MEX3D | 13.20700 | 19.07750 | 0.53057 | 1.52978 | 1.05E-03 | 3.50E-02 | yes |
| PLBD1 | 28.33950 | 40.93390 | 0.53048 | 1.58083 | 4.50E-04 | 1.75E-02 | yes |
| BRIX1 | 44.78640 | 64.63830 | 0.52933 | 1.58777 | 2.50E-04 | 1.07E-02 | yes |
| KIF13A | 19.96080 | 28.77540 | 0.52767 | 1.43987 | 1.45E-03 | 4.50E-02 | yes |
| DKK1 | 94.15230 | 135.65500 | 0.52688 | 1.53556 | 4.50E-04 | 1.75E-02 | yes |
| TUBA1A | 173.52400 | 249.65300 | 0.52479 | 1.64282 | 1.50E-04 | 7.06E-03 | yes |
| GATA2 | 31.39620 | 45.12160 | 0.52323 | 1.57082 | 2.50E-04 | 1.07E-02 | yes |
| FAM214B | 18.08510 | 25.98600 | 0.52293 | 1.57202 | 2.50E-04 | 1.07E-02 | yes |
| GSE1 | 6.88910 | 9.89813 | 0.52284 | 1.57605 | 4.50E-04 | 1.75E-02 | yes |
| NCK2 | 15.52620 | 22.26390 | 0.52000 | 1.46736 | 1.10E-03 | 3.61E-02 | yes |
| CBLB | 9.30141 | 13.32330 | 0.51843 | 1.45196 | 1.10E-03 | 3.61E-02 | yes |
| FAM89B | 27.81140 | 39.60580 | 0.51004 | 1.45567 | 9.50E-04 | 3.18E-02 | yes |
| KRT18 | 205.07300 | 290.83500 | 0.50407 | 1.41831 | 1.30E-03 | 4.16E-02 | yes |
| PPFIBP1 | 12.75910 | 18.03630 | 0.49938 | 1.58502 | 5.50E-04 | 2.07E-02 | yes |
| GULP1 | 19.41500 | 27.44070 | 0.49915 | 1.54148 | 4.50E-04 | 1.75E-02 | yes |
| CCDC71L | 18.37100 | 25.94790 | 0.49819 | 1.57170 | 4.50E-04 | 1.75E-02 | yes |
| PLAT | 102.74000 | 145.02200 | 0.49727 | 1.52210 | 8.50E-04 | 2.94E-02 | yes |
| C11orf95 | 7.47816 | 10.52270 | 0.49275 | 1.44617 | 1.50E-03 | 4.63E-02 | yes |
| COL11A1 | 17.73740 | 24.94480 | 0.49195 | 1.48736 | 7.00E-04 | 2.48E-02 | yes |
| ZNF503 | 21.90420 | 30.80010 | 0.49173 | 1.49500 | 1.10E-03 | 3.61E-02 | yes |
| TLR4 | 7.49692 | 10.53810 | 0.49125 | 1.42607 | 1.40E-03 | 4.38E-02 | yes |
| CAP2 | 35.32100 | 49.62720 | 0.49061 | 1.58586 | 2.50E-04 | 1.07E-02 | yes |
| ALPK2 | 7.53447 | 10.58200 | 0.49004 | 1.44773 | 8.50E-04 | 2.94E-02 | yes |
| AMIGO2 | 57.36540 | 80.24110 | 0.48416 | 1.48914 | 6.00E-04 | 2.21E-02 | yes |
| HIPK2 | 3.25477 | 4.54812 | 0.48271 | 1.44243 | 1.30E-03 | 4.16E-02 | yes |
| CMTM6 | 44.55020 | 62.25300 | 0.48271 | 1.58313 | 3.00E-04 | 1.25E-02 | yes |
| TP53INP1 | 10.81470 | 15.04870 | 0.47665 | 1.44964 | 1.40E-03 | 4.38E-02 | yes |
| VCAN | 31.03050 | 43.16520 | 0.47619 | 1.40457 | 1.50E-03 | 4.63E-02 | yes |
| PCGF3 | 11.28590 | 15.64740 | 0.47140 | 1.47463 | 9.50E-04 | 3.18E-02 | yes |
| YPEL5 | 61.08770 | 84.63770 | 0.47042 | 1.49564 | 7.00E-04 | 2.48E-02 | yes |
| DSE | 25.88120 | 35.64110 | 0.46164 | 1.49024 | 8.50E-04 | 2.94E-02 | yes |
| LIMK1 | 36.87410 | 50.63120 | 0.45742 | 1.48360 | 6.00E-04 | 2.21E-02 | yes |
| FURIN | 44.70920 | 60.73360 | 0.44192 | 1.43990 | 1.45E-03 | 4.50E-02 | yes |
| MAN2A1 | 37.69590 | 28.15140 | -0.42120 | -1.37168 | 1.65E-03 | 4.96E-02 | yes |
| CYB5B | 57.70060 | 42.81170 | -0.43058 | -1.39348 | 1.65E-03 | 4.96E-02 | yes |
| S100A10 | 105.81100 | 77.55910 | -0.44812 | -1.38204 | 1.60E-03 | 4.84E-02 | yes |
| ADM | 187.72000 | 137.20500 | -0.45225 | -1.46864 | 9.50E-04 | 3.18E-02 | yes |
| GLT8D2 | 36.79800 | 26.63850 | -0.46612 | -1.36473 | 1.65E-03 | 4.96E-02 | yes |
| SMAD3 | 24.37090 | 17.63420 | -0.46679 | -1.47023 | 1.10E-03 | 3.61E-02 | yes |
| EML1 | 19.07960 | 13.74220 | -0.47342 | -1.45466 | 1.50E-03 | 4.63E-02 | yes |
| SEMA3A | 17.94940 | 12.87940 | -0.47888 | -1.44960 | 1.10E-03 | 3.61E-02 | yes |
| CD9 | 145.76200 | 104.44800 | -0.48083 | -1.55529 | 3.50E-04 | 1.42E-02 | yes |
| RAD23B | 84.93860 | 60.48770 | -0.48978 | -1.57158 | 4.50E-04 | 1.75E-02 | yes |
| PEG10 | 31.64670 | 22.46980 | -0.49407 | -1.60302 | 6.50E-04 | 2.35E-02 | yes |
| NEFM | 43.99600 | 31.22570 | -0.49464 | -1.33778 | 1.30E-03 | 4.16E-02 | yes |
| DNMBP | 14.72900 | 10.45370 | -0.49465 | -1.55154 | 6.50E-04 | 2.35E-02 | yes |
| COL7A1 | 81.92910 | 57.97910 | -0.49884 | -1.43907 | 9.00E-04 | 3.09E-02 | yes |
| CLDN11 | 55.73430 | 39.43840 | -0.49896 | -1.44420 | 1.55E-03 | 4.75E-02 | yes |
| ETS2 | 28.23060 | 19.93530 | -0.50194 | -1.55773 | 7.00E-04 | 2.48E-02 | yes |
| GFRA1 | 6.90656 | 4.85936 | -0.50720 | -1.41303 | 1.30E-03 | 4.16E-02 | yes |
| POU2F2 | 16.51160 | 11.60440 | -0.50882 | -1.38576 | 1.60E-03 | 4.84E-02 | yes |
| STARD13 | 10.19160 | 7.13427 | -0.51454 | -1.50033 | 7.00E-04 | 2.48E-02 | yes |
| TNFRSF1A | 43.77950 | 30.64260 | -0.51472 | -1.57840 | 8.50E-04 | 2.94E-02 | yes |
| USP53 | 63.20720 | 44.10720 | -0.51907 | -1.58548 | 1.00E-04 | 5.02E-03 | yes |
| LINC00152 | 221.70900 | 154.69300 | -0.51926 | -1.54558 | 5.50E-04 | 2.07E-02 | yes |
| JAZF1 | 28.18170 | 19.50290 | -0.53107 | -1.64533 | 1.00E-04 | 5.02E-03 | yes |
| HMGA1 | 171.73100 | 118.36700 | -0.53688 | -1.70581 | 1.00E-04 | 5.02E-03 | yes |
| MAN1A1 | 46.64770 | 32.07790 | -0.54023 | -1.66219 | 2.50E-04 | 1.07E-02 | yes |
| SQRDL | 22.71380 | 15.56270 | -0.54548 | -1.45955 | 1.20E-03 | 3.89E-02 | yes |
| OSBPL3 | 10.62310 | 7.27546 | -0.54609 | -1.64481 | 3.00E-04 | 1.25E-02 | yes |
| TMTC1 | 13.92500 | 9.48789 | -0.55352 | -1.76348 | 1.50E-04 | 7.06E-03 | yes |
| IL6 | 52.62620 | 35.71840 | -0.55911 | -1.44619 | 1.35E-03 | 4.28E-02 | yes |
| CPT1A | 18.92840 | 12.84470 | -0.55938 | -1.68448 | 4.00E-04 | 1.61E-02 | yes |
| RRM2 | 34.43610 | 23.31740 | -0.56251 | -1.60899 | 4.50E-04 | 1.75E-02 | yes |
| ARRDC4 | 13.70770 | 9.22897 | -0.57075 | -1.62480 | 2.00E-04 | 8.81E-03 | yes |
| NEFL | 39.02810 | 26.27360 | -0.57090 | -1.51364 | 6.50E-04 | 2.35E-02 | yes |
| LIPA | 129.07700 | 86.77470 | -0.57289 | -1.81325 | 5.00E-05 | 2.76E-03 | yes |
| MGLL | 95.89750 | 64.43730 | -0.57360 | -1.78739 | 2.50E-04 | 1.07E-02 | yes |
| WNT5A | 170.40200 | 114.45600 | -0.57416 | -1.52972 | 5.50E-04 | 2.07E-02 | yes |
| ZFP36L2 | 35.81750 | 24.05590 | -0.57427 | -1.81732 | 5.00E-05 | 2.76E-03 | yes |
| FAM129A | 7.37930 | 4.94925 | -0.57627 | -1.63541 | 3.00E-04 | 1.25E-02 | yes |
| JADE1 | 7.64404 | 5.12503 | -0.57677 | -1.49880 | 1.20E-03 | 3.89E-02 | yes |
| EFNB2 | 7.38820 | 4.92044 | -0.58644 | -1.46543 | 1.35E-03 | 4.28E-02 | yes |
| TMEM158 | 39.49370 | 26.20060 | -0.59202 | -1.70762 | 1.50E-04 | 7.06E-03 | yes |
| PKIG | 161.23400 | 106.75500 | -0.59486 | -1.91646 | 1.00E-04 | 5.02E-03 | yes |
| PTPRF | 16.70090 | 11.03170 | -0.59828 | -1.90312 | 5.00E-05 | 2.76E-03 | yes |
| SNTB1 | 9.89692 | 6.51710 | -0.60275 | -1.65456 | 1.50E-04 | 7.06E-03 | yes |
| TNFSF4 | 30.30880 | 19.87580 | -0.60873 | -1.87234 | 5.00E-05 | 2.76E-03 | yes |
| LIMA1 | 88.73700 | 58.02900 | -0.61276 | -1.91737 | 5.00E-05 | 2.76E-03 | yes |
| KRTAP1-5 | 37.80110 | 24.67570 | -0.61534 | -1.56442 | 6.00E-04 | 2.21E-02 | yes |
| CEBPD | 40.74050 | 26.38940 | -0.62650 | -1.71223 | 1.00E-04 | 5.02E-03 | yes |
| GJA1 | 318.79800 | 206.13100 | -0.62908 | -1.70290 | 5.00E-05 | 2.76E-03 | yes |
| FAM65C | 12.72800 | 8.21985 | -0.63082 | -1.74911 | 1.00E-04 | 5.02E-03 | yes |
| GRK5 | 17.17940 | 11.09310 | -0.63101 | -1.70232 | 1.00E-04 | 5.02E-03 | yes |
| UCK2 | 11.41010 | 7.35540 | -0.63343 | -1.81829 | 1.00E-04 | 5.02E-03 | yes |
| THSD4 | 15.79380 | 10.16340 | -0.63598 | -2.03920 | 5.00E-05 | 2.76E-03 | yes |
| CHST15 | 6.49844 | 4.18039 | -0.63646 | -1.55899 | 4.50E-04 | 1.75E-02 | yes |
| COL6A1 | 336.46200 | 215.43300 | -0.64320 | -1.46611 | 1.40E-03 | 4.38E-02 | yes |
| DOCK11 | 8.29663 | 5.30039 | -0.64643 | -1.83910 | 5.00E-05 | 2.76E-03 | yes |
| SLFN11 | 5.84352 | 3.73299 | -0.64651 | -1.56296 | 4.50E-04 | 1.75E-02 | yes |
| AOX1 | 13.31640 | 8.47665 | -0.65164 | -1.88815 | 5.00E-05 | 2.76E-03 | yes |
| UGCG | 35.69550 | 22.69710 | -0.65324 | -2.03311 | 5.00E-05 | 2.76E-03 | yes |
| NPY1R | 8.92166 | 5.67236 | -0.65336 | -1.50133 | 5.50E-04 | 2.07E-02 | yes |
| NID2 | 33.78500 | 21.45660 | -0.65496 | -2.06462 | 5.00E-05 | 2.76E-03 | yes |
| SLC2A1 | 68.46810 | 43.42820 | -0.65680 | -2.09039 | 5.00E-05 | 2.76E-03 | yes |
| RIN1 | 12.95260 | 8.21309 | -0.65725 | -1.66524 | 2.00E-04 | 8.81E-03 | yes |
| PCSK9 | 8.83122 | 5.59274 | -0.65906 | -1.56720 | 5.50E-04 | 2.07E-02 | yes |
| PRDM8 | 7.11197 | 4.50252 | -0.65952 | -1.52682 | 5.00E-04 | 1.93E-02 | yes |
| GLRX | 366.78800 | 232.16700 | -0.65978 | -2.06442 | 5.00E-05 | 2.76E-03 | yes |
| SIPA1 | 16.74430 | 10.58220 | -0.66203 | -1.89935 | 5.00E-05 | 2.76E-03 | yes |
| ATP2B1 | 80.52940 | 50.77910 | -0.66528 | -2.02477 | 5.00E-05 | 2.76E-03 | yes |
| LRRC8C | 5.71218 | 3.58076 | -0.67378 | -1.80841 | 5.00E-05 | 2.76E-03 | yes |
| NMT2 | 18.71580 | 11.72020 | -0.67527 | -1.82374 | 1.00E-04 | 5.02E-03 | yes |
| KRT19 | 363.98100 | 227.31400 | -0.67918 | -1.98970 | 5.00E-05 | 2.76E-03 | yes |
| SH3RF3 | 7.74250 | 4.80309 | -0.68884 | -1.75909 | 5.00E-05 | 2.76E-03 | yes |
| PMAIP1 | 33.35870 | 20.48140 | -0.70375 | -2.01675 | 5.00E-05 | 2.76E-03 | yes |
| BDKRB2 | 40.63410 | 24.77180 | -0.71399 | -2.27009 | 5.00E-05 | 2.76E-03 | yes |
| ADAMTS12 | 8.77622 | 5.34739 | -0.71476 | -1.76417 | 3.00E-04 | 1.25E-02 | yes |
| MGAT5 | 72.05020 | 43.81590 | -0.71755 | -2.19209 | 5.00E-05 | 2.76E-03 | yes |
| TUFT1 | 6.68260 | 4.04919 | -0.72277 | -1.53799 | 9.50E-04 | 3.18E-02 | yes |
| PTGS2 | 5.08476 | 3.06696 | -0.72937 | -1.59998 | 6.00E-04 | 2.21E-02 | yes |
| SLC22A4 | 18.49190 | 11.14980 | -0.72988 | -1.74745 | 1.00E-04 | 5.02E-03 | yes |
| FAM124A | 5.59443 | 3.36653 | -0.73273 | -1.70510 | 4.50E-04 | 1.75E-02 | yes |
| F3 | 72.21490 | 43.34530 | -0.73642 | -2.23474 | 5.00E-05 | 2.76E-03 | yes |
| LPPR4 | 3.77640 | 2.25610 | -0.74318 | -1.52807 | 1.35E-03 | 4.28E-02 | yes |
| SDPR | 8.66546 | 5.15037 | -0.75060 | -1.76203 | 5.00E-05 | 2.76E-03 | yes |
| NDRG1 | 36.48640 | 21.66930 | -0.75171 | -2.33735 | 5.00E-05 | 2.76E-03 | yes |
| PTGS1 | 8.02641 | 4.76343 | -0.75275 | -1.84446 | 1.00E-04 | 5.02E-03 | yes |
| MTUS1 | 4.52431 | 2.67029 | -0.76070 | -1.48347 | 9.50E-04 | 3.18E-02 | yes |
| FJX1 | 121.91500 | 71.80440 | -0.76373 | -2.37098 | 5.00E-05 | 2.76E-03 | yes |
| DUSP5 | 15.37410 | 9.04897 | -0.76467 | -1.94719 | 1.00E-04 | 5.02E-03 | yes |
| TFAP2C | 16.46800 | 9.64494 | -0.77182 | -1.99844 | 5.00E-05 | 2.76E-03 | yes |
| PELI1 | 4.81009 | 2.81664 | -0.77209 | -1.55464 | 8.00E-04 | 2.82E-02 | yes |
| TNFRSF21 | 56.35940 | 32.89030 | -0.77699 | -2.50050 | 5.00E-05 | 2.76E-03 | yes |
| STAMBPL1 | 30.80850 | 17.97210 | -0.77757 | -2.22540 | 5.00E-05 | 2.76E-03 | yes |
| ERMP1 | 38.26810 | 22.26210 | -0.78156 | -2.39448 | 5.00E-05 | 2.76E-03 | yes |
| CXCL3 | 22.41700 | 12.99350 | -0.78680 | -1.69541 | 2.00E-04 | 8.81E-03 | yes |
| NRXN3 | 2.40763 | 1.38758 | -0.79504 | -1.71065 | 2.00E-04 | 8.81E-03 | yes |
| HIC1 | 20.10190 | 11.48420 | -0.80768 | -2.32789 | 5.00E-05 | 2.76E-03 | yes |
| EFNA5 | 3.48450 | 1.97985 | -0.81556 | -1.64902 | 8.00E-04 | 2.82E-02 | yes |
| PDGFRL | 8.40424 | 4.76950 | -0.81728 | -1.54182 | 6.00E-04 | 2.21E-02 | yes |
| SLIT2 | 3.98379 | 2.25815 | -0.81900 | -1.77706 | 1.00E-04 | 5.02E-03 | yes |
| FABP3 | 26.07430 | 14.70110 | -0.82670 | -1.75812 | 2.00E-04 | 8.81E-03 | yes |
| AIM1 | 3.40181 | 1.91111 | -0.83189 | -1.79346 | 5.00E-05 | 2.76E-03 | yes |
| FBLN1 | 131.58400 | 73.89770 | -0.83238 | -2.29799 | 5.00E-05 | 2.76E-03 | yes |
| KLHL29 | 4.76853 | 2.66685 | -0.83841 | -1.86268 | 5.00E-05 | 2.76E-03 | yes |
| SH2D5 | 4.91886 | 2.74597 | -0.84101 | -1.69574 | 3.00E-04 | 1.25E-02 | yes |
| CTNNAL1 | 37.00980 | 20.56670 | -0.84760 | -2.61513 | 5.00E-05 | 2.76E-03 | yes |
| CPA4 | 16.64980 | 9.24082 | -0.84941 | -2.14232 | 5.00E-05 | 2.76E-03 | yes |
| SERPINB2 | 57.09970 | 31.65550 | -0.85103 | -2.30847 | 5.00E-05 | 2.76E-03 | yes |
| IMPA2 | 8.98567 | 4.96532 | -0.85574 | -1.52906 | 1.05E-03 | 3.50E-02 | yes |
| ADGRG6 | 11.71000 | 6.46766 | -0.85642 | -2.55640 | 5.00E-05 | 2.76E-03 | yes |
| NPAS2 | 6.12877 | 3.37299 | -0.86157 | -1.86982 | 2.00E-04 | 8.81E-03 | yes |
| MRGPRF | 30.45520 | 16.73020 | -0.86423 | -2.46163 | 5.00E-05 | 2.76E-03 | yes |
| STRA6 | 28.30440 | 15.46490 | -0.87202 | -2.54760 | 5.00E-05 | 2.76E-03 | yes |
| RUNX1T1 | 3.52012 | 1.91394 | -0.87908 | -1.93846 | 5.00E-05 | 2.76E-03 | yes |
| PTCH1 | 1.51557 | 0.82096 | -0.88448 | -1.54604 | 4.50E-04 | 1.75E-02 | yes |
| TMEM119 | 39.51240 | 21.31450 | -0.89047 | -2.51948 | 5.00E-05 | 2.76E-03 | yes |
| DCHS1 | 3.81062 | 2.05342 | -0.89200 | -2.25464 | 5.00E-05 | 2.76E-03 | yes |
| PTX3 | 110.23700 | 58.99590 | -0.90192 | -2.74361 | 5.00E-05 | 2.76E-03 | yes |
| LRRN4CL | 8.99622 | 4.80369 | -0.90518 | -1.94902 | 5.00E-05 | 2.76E-03 | yes |
| CORO2B | 7.81280 | 4.12849 | -0.92023 | -2.06845 | 5.00E-05 | 2.76E-03 | yes |
| GAS1 | 11.99060 | 6.31599 | -0.92483 | -2.13586 | 5.00E-05 | 2.76E-03 | yes |
| SLC38A4 | 3.42490 | 1.80086 | -0.92738 | -1.63846 | 5.50E-04 | 2.07E-02 | yes |
| C17orf58 | 6.79519 | 3.56840 | -0.92924 | -1.48328 | 1.60E-03 | 4.84E-02 | yes |
| CAV1 | 274.67100 | 143.97600 | -0.93188 | -2.75336 | 5.00E-05 | 2.76E-03 | yes |
| DUSP6 | 36.47650 | 19.11360 | -0.93237 | -2.80621 | 5.00E-05 | 2.76E-03 | yes |
| CHST2 | 50.25540 | 26.30420 | -0.93399 | -2.99897 | 5.00E-05 | 2.76E-03 | yes |
| ALDH1A1 | 7.73656 | 4.04540 | -0.93541 | -1.78114 | 1.50E-04 | 7.06E-03 | yes |
| IL1B | 14.57400 | 7.57056 | -0.94493 | -1.66125 | 3.50E-04 | 1.42E-02 | yes |
| ARHGAP29 | 10.81020 | 5.57870 | -0.95440 | -2.87286 | 5.00E-05 | 2.76E-03 | yes |
| SVEP1 | 8.10741 | 4.16481 | -0.96099 | -2.76849 | 5.00E-05 | 2.76E-03 | yes |
| GPRC5A | 39.76440 | 20.29070 | -0.97066 | -2.95002 | 5.00E-05 | 2.76E-03 | yes |
| PDGFD | 7.33116 | 3.73964 | -0.97114 | -2.22270 | 5.00E-05 | 2.76E-03 | yes |
| FAM65B | 2.52044 | 1.26890 | -0.99010 | -1.54482 | 9.00E-04 | 3.09E-02 | yes |
| LINC01085 | 10.09160 | 5.01917 | -1.00763 | -2.00323 | 5.00E-05 | 2.76E-03 | yes |
| NTN1 | 1.92509 | 0.94343 | -1.02894 | -1.62699 | 7.00E-04 | 2.48E-02 | yes |
| ATP8B1 | 8.84069 | 4.29314 | -1.04213 | -2.76767 | 5.00E-05 | 2.76E-03 | yes |
| ATF3 | 9.23069 | 4.42171 | -1.06183 | -2.05809 | 5.00E-05 | 2.76E-03 | yes |
| MEST | 153.78900 | 73.43300 | -1.06645 | -2.61620 | 5.00E-05 | 2.76E-03 | yes |
| CREB5 | 1.12378 | 0.53614 | -1.06769 | -1.67041 | 5.50E-04 | 2.07E-02 | yes |
| ACPP | 4.96051 | 2.35864 | -1.07253 | -1.73174 | 3.50E-04 | 1.42E-02 | yes |
| WISP1 | 6.77887 | 3.22205 | -1.07307 | -2.20454 | 5.00E-05 | 2.76E-03 | yes |
| MEOX1 | 4.54219 | 2.14144 | -1.08481 | -1.64702 | 9.50E-04 | 3.18E-02 | yes |
| ABCA3 | 1.69444 | 0.79658 | -1.08891 | -1.82390 | 5.00E-05 | 2.76E-03 | yes |
| KRT34 | 55.31460 | 25.89540 | -1.09497 | -3.10806 | 5.00E-05 | 2.76E-03 | yes |
| CCBE1 | 1.56130 | 0.72205 | -1.11258 | -1.76216 | 4.50E-04 | 1.75E-02 | yes |
| SDC4 | 54.58920 | 25.09090 | -1.12145 | -3.39524 | 5.00E-05 | 2.76E-03 | yes |
| COL5A3 | 2.79802 | 1.27101 | -1.13843 | -1.85155 | 1.00E-04 | 5.02E-03 | yes |
| TNFRSF19 | 27.94740 | 12.58770 | -1.15070 | -3.30842 | 5.00E-05 | 2.76E-03 | yes |
| HLX | 14.08640 | 6.20194 | -1.18351 | -2.70232 | 5.00E-05 | 2.76E-03 | yes |
| C10orf54 | 4.05626 | 1.78091 | -1.18753 | -2.32754 | 5.00E-05 | 2.76E-03 | yes |
| CRABP2 | 25.74620 | 11.25320 | -1.19402 | -2.09758 | 5.00E-05 | 2.76E-03 | yes |
| AFAP1L2 | 2.63455 | 1.15069 | -1.19505 | -1.92390 | 5.00E-05 | 2.76E-03 | yes |
| SOCS1 | 16.86980 | 7.33814 | -1.20096 | -2.30243 | 5.00E-05 | 2.76E-03 | yes |
| H19 | 3.94165 | 1.68951 | -1.22220 | -1.27644 | 9.50E-04 | 3.18E-02 | yes |
| DPT | 2.80901 | 1.19188 | -1.23682 | -1.43848 | 1.60E-03 | 4.84E-02 | yes |
| PRELP | 1.53562 | 0.64920 | -1.24209 | -1.86203 | 5.00E-05 | 2.76E-03 | yes |
| CCDC109B | 5.42082 | 2.29133 | -1.24233 | -1.64021 | 1.35E-03 | 4.28E-02 | yes |
| BMP4 | 17.99420 | 7.58092 | -1.24708 | -2.70013 | 5.00E-05 | 2.76E-03 | yes |
| DENND2A | 14.43270 | 5.97355 | -1.27268 | -3.18078 | 5.00E-05 | 2.76E-03 | yes |
| ADORA2B | 4.57041 | 1.88078 | -1.28099 | -1.82032 | 1.50E-04 | 7.06E-03 | yes |
| RAMP1 | 40.59850 | 16.61540 | -1.28891 | -2.83504 | 5.00E-05 | 2.76E-03 | yes |
| DLL4 | 7.19488 | 2.86610 | -1.32788 | -2.68726 | 5.00E-05 | 2.76E-03 | yes |
| COL14A1 | 12.72610 | 5.00492 | -1.34637 | -3.26241 | 5.00E-05 | 2.76E-03 | yes |
| SFRP1 | 39.48030 | 15.50240 | -1.34864 | -3.52310 | 5.00E-05 | 2.76E-03 | yes |
| CCL2 | 130.55900 | 51.25690 | -1.34888 | -3.53267 | 5.00E-05 | 2.76E-03 | yes |
| TMEM171 | 8.67909 | 3.40010 | -1.35197 | -2.06091 | 5.00E-05 | 2.76E-03 | yes |
| HGF | 3.10789 | 1.20111 | -1.37156 | -1.69990 | 5.00E-04 | 1.93E-02 | yes |
| TGFBR3 | 2.07059 | 0.78805 | -1.39369 | -2.26342 | 5.00E-05 | 2.76E-03 | yes |
| CHODL | 0.89094 | 0.32750 | -1.44386 | -1.36651 | 1.40E-03 | 4.38E-02 | yes |
| COLEC12 | 1.71079 | 0.62814 | -1.44549 | -1.62145 | 1.25E-03 | 4.04E-02 | yes |
| RASGRP2 | 1.88960 | 0.69271 | -1.44775 | -1.71816 | 2.00E-04 | 8.81E-03 | yes |
| SYT7 | 1.84187 | 0.65369 | -1.49451 | -2.25339 | 5.00E-05 | 2.76E-03 | yes |
| TRPC6 | 2.71689 | 0.95213 | -1.51273 | -2.44215 | 5.00E-05 | 2.76E-03 | yes |
| TNFSF18 | 33.19580 | 11.30570 | -1.55395 | -2.75992 | 5.00E-05 | 2.76E-03 | yes |
| LOXL4 | 19.00850 | 5.97911 | -1.66864 | -4.37867 | 5.00E-05 | 2.76E-03 | yes |
| ALDH1A3 | 8.65760 | 2.50424 | -1.78959 | -3.57808 | 5.00E-05 | 2.76E-03 | yes |
| RGCC | 43.83240 | 11.92000 | -1.87861 | -4.19227 | 5.00E-05 | 2.76E-03 | yes |
| HSD17B2 | 26.67470 | 6.67015 | -1.99968 | -3.87055 | 5.00E-05 | 2.76E-03 | yes |
| NANOS1 | 8.16068 | 1.11310 | -2.87410 | -5.20858 | 5.00E-05 | 2.76E-03 | yes |
