# Supplementary material for: Dysregulated BMP2 in the Placenta May Contribute to Early-Onset Preeclampsia by Regulating Human Trophoblast Expression of Extracellular Matrix and Adhesion Molecules
Source: Front Cell Dev Biol. 2021 Dec 14;9:768669. doi: 10.3389/fcell.2021.768669 (PMC8712873; doi:10.3389/fcell.2021.768669)
Supplement: Supplementary file 4 [file Table4.docx]

| **Supplementary Table 4. Top 50 GO annotations enriched among BMP2 regulated genes** | | | | | | | | |
| --- | --- | --- | --- | --- | --- | --- | --- | --- |
| ONTOLOGY | ID | Description | GeneRatio | BgRatio | pvalue | p.adjust | qvalue | Count |
| BP | GO:0030198 | extracellular matrix organization | 37/241 | 405/21265 | 7.01E-23 | 4.00E-19 | 2.80E-19 | 37 |
| BP | GO:0007155 | cell adhesion | 67/241 | 1568/21265 | 5.03E-22 | 1.22E-18 | 8.55E-19 | 67 |
| BP | GO:0022610 | biological adhesion | 67/241 | 1575/21265 | 6.42E-22 | 1.22E-18 | 8.55E-19 | 67 |
| BP | GO:0043062 | extracellular structure organization | 38/241 | 465/21265 | 9.13E-22 | 1.30E-18 | 9.13E-19 | 38 |
| BP | GO:0009887 | animal organ morphogenesis | 52/241 | 1146/21265 | 4.59E-18 | 5.23E-15 | 3.67E-15 | 52 |
| BP | GO:0030155 | regulation of cell adhesion | 43/241 | 794/21265 | 9.94E-18 | 9.45E-15 | 6.62E-15 | 43 |
| BP | GO:0042127 | regulation of cell proliferation | 65/241 | 1840/21265 | 4.45E-17 | 3.63E-14 | 2.54E-14 | 65 |
| BP | GO:0008285 | negative regulation of cell proliferation | 42/241 | 834/21265 | 3.31E-16 | 2.36E-13 | 1.66E-13 | 42 |
| BP | GO:0035295 | tube development | 49/241 | 1173/21265 | 1.33E-15 | 8.45E-13 | 5.92E-13 | 49 |
| BP | GO:0060429 | epithelium development | 54/241 | 1416/21265 | 1.66E-15 | 9.50E-13 | 6.65E-13 | 54 |
| BP | GO:0016477 | cell migration | 58/241 | 1621/21265 | 1.98E-15 | 1.03E-12 | 7.20E-13 | 58 |
| BP | GO:0009611 | response to wounding | 38/241 | 721/21265 | 2.39E-15 | 1.14E-12 | 7.98E-13 | 38 |
| BP | GO:0048870 | cell motility | 60/241 | 1774/21265 | 7.29E-15 | 2.97E-12 | 2.08E-12 | 60 |
| BP | GO:0051674 | localization of cell | 60/241 | 1774/21265 | 7.29E-15 | 2.97E-12 | 2.08E-12 | 60 |
| MF | GO:0038062 | protein tyrosine kinase collagen receptor activity | 7/241 | 8/21265 | 1.74E-13 | 6.64E-11 | 4.65E-11 | 7 |
| BP | GO:0007167 | enzyme linked receptor protein signaling pathway | 44/241 | 1099/21265 | 2.10E-13 | 7.51E-11 | 5.26E-11 | 44 |
| BP | GO:0097435 | supramolecular fiber organization | 35/241 | 714/21265 | 2.84E-13 | 9.53E-11 | 6.68E-11 | 35 |
| BP | GO:0035239 | tube morphogenesis | 40/241 | 970/21265 | 1.22E-12 | 3.87E-10 | 2.71E-10 | 40 |
| BP | GO:0042060 | wound healing | 31/241 | 596/21265 | 1.61E-12 | 4.82E-10 | 3.38E-10 | 31 |
| BP | GO:1903053 | regulation of extracellular matrix organization | 11/241 | 45/21265 | 2.28E-12 | 6.50E-10 | 4.56E-10 | 11 |
| BP | GO:0022008 | neurogenesis | 54/241 | 1715/21265 | 3.86E-12 | 1.05E-09 | 7.34E-10 | 54 |
| BP | GO:0048646 | anatomical structure formation involved in morphogenesis | 45/241 | 1264/21265 | 6.14E-12 | 1.59E-09 | 1.12E-09 | 45 |
| BP | GO:0061302 | smooth muscle cell-matrix adhesion | 7/241 | 11/21265 | 6.99E-12 | 1.73E-09 | 1.22E-09 | 7 |
| CC | GO:0031012 | extracellular matrix | 29/241 | 562/21265 | 1.08E-11 | 2.57E-09 | 1.80E-09 | 29 |
| BP | GO:0048699 | generation of neurons | 51/241 | 1605/21265 | 1.26E-11 | 2.89E-09 | 2.02E-09 | 51 |
| BP | GO:0042981 | regulation of apoptotic process | 53/241 | 1716/21265 | 1.33E-11 | 2.92E-09 | 2.05E-09 | 53 |
| BP | GO:0072359 | circulatory system development | 43/241 | 1201/21265 | 1.64E-11 | 3.46E-09 | 2.43E-09 | 43 |
| BP | GO:0060443 | mammary gland morphogenesis | 11/241 | 54/21265 | 1.96E-11 | 4.01E-09 | 2.81E-09 | 11 |
| BP | GO:0048729 | tissue morphogenesis | 33/241 | 745/21265 | 2.21E-11 | 4.35E-09 | 3.05E-09 | 33 |
| CC | GO:0062023 | collagen-containing extracellular matrix | 25/241 | 430/21265 | 2.53E-11 | 4.69E-09 | 3.29E-09 | 25 |
| BP | GO:0043067 | regulation of programmed cell death | 53/241 | 1748/21265 | 2.66E-11 | 4.69E-09 | 3.29E-09 | 53 |
| BP | GO:0051094 | positive regulation of developmental process | 49/241 | 1531/21265 | 2.71E-11 | 4.69E-09 | 3.29E-09 | 49 |
| MF | GO:0038064 | collagen receptor activity | 8/241 | 20/21265 | 2.71E-11 | 4.69E-09 | 3.29E-09 | 8 |
| BP | GO:0022617 | extracellular matrix disassembly | 13/241 | 92/21265 | 3.72E-11 | 6.25E-09 | 4.38E-09 | 13 |
| BP | GO:0010715 | regulation of extracellular matrix disassembly | 8/241 | 21/21265 | 4.34E-11 | 7.08E-09 | 4.96E-09 | 8 |
| BP | GO:0031589 | cell-substrate adhesion | 23/241 | 373/21265 | 5.14E-11 | 8.16E-09 | 5.71E-09 | 23 |
| BP | GO:0022612 | gland morphogenesis | 15/241 | 139/21265 | 5.93E-11 | 9.15E-09 | 6.41E-09 | 15 |
| BP | GO:0043065 | positive regulation of apoptotic process | 32/241 | 738/21265 | 7.90E-11 | 1.19E-08 | 8.31E-09 | 32 |
| BP | GO:0010941 | regulation of cell death | 55/241 | 1922/21265 | 9.33E-11 | 1.37E-08 | 9.56E-09 | 55 |
| BP | GO:0045597 | positive regulation of cell differentiation | 39/241 | 1069/21265 | 9.60E-11 | 1.37E-08 | 9.60E-09 | 39 |
| BP | GO:0030182 | neuron differentiation | 46/241 | 1429/21265 | 1.03E-10 | 1.41E-08 | 9.89E-09 | 46 |
| BP | GO:0043068 | positive regulation of programmed cell death | 32/241 | 746/21265 | 1.04E-10 | 1.41E-08 | 9.89E-09 | 32 |
| BP | GO:0048666 | neuron development | 41/241 | 1174/21265 | 1.12E-10 | 1.49E-08 | 1.04E-08 | 41 |
| BP | GO:0040007 | growth | 38/241 | 1027/21265 | 1.15E-10 | 1.50E-08 | 1.05E-08 | 38 |
| BP | GO:0010942 | positive regulation of cell death | 33/241 | 799/21265 | 1.38E-10 | 1.74E-08 | 1.22E-08 | 33 |
| BP | GO:0060749 | mammary gland alveolus development | 8/241 | 24/21265 | 1.52E-10 | 1.85E-08 | 1.30E-08 | 8 |
| BP | GO:0061377 | mammary gland lobule development | 8/241 | 24/21265 | 1.52E-10 | 1.85E-08 | 1.30E-08 | 8 |
| BP | GO:0032989 | cellular component morphogenesis | 41/241 | 1190/21265 | 1.69E-10 | 1.97E-08 | 1.38E-08 | 41 |
| BP | GO:0048585 | negative regulation of response to stimulus | 54/241 | 1895/21265 | 1.69E-10 | 1.97E-08 | 1.38E-08 | 54 |
| BP | GO:0030334 | regulation of cell migration | 37/241 | 998/21265 | 1.98E-10 | 2.26E-08 | 1.59E-08 | 37 |
