# Supplementary material for: Dysregulated BMP2 in the Placenta May Contribute to Early-Onset Preeclampsia by Regulating Human Trophoblast Expression of Extracellular Matrix and Adhesion Molecules
Source: Front Cell Dev Biol. 2021 Dec 14;9:768669. doi: 10.3389/fcell.2021.768669 (PMC8712873; doi:10.3389/fcell.2021.768669)
Supplement: Supplementary file 5 [file DataSheet1.DOCX]

**Supplementary Figures and Figure Legends**


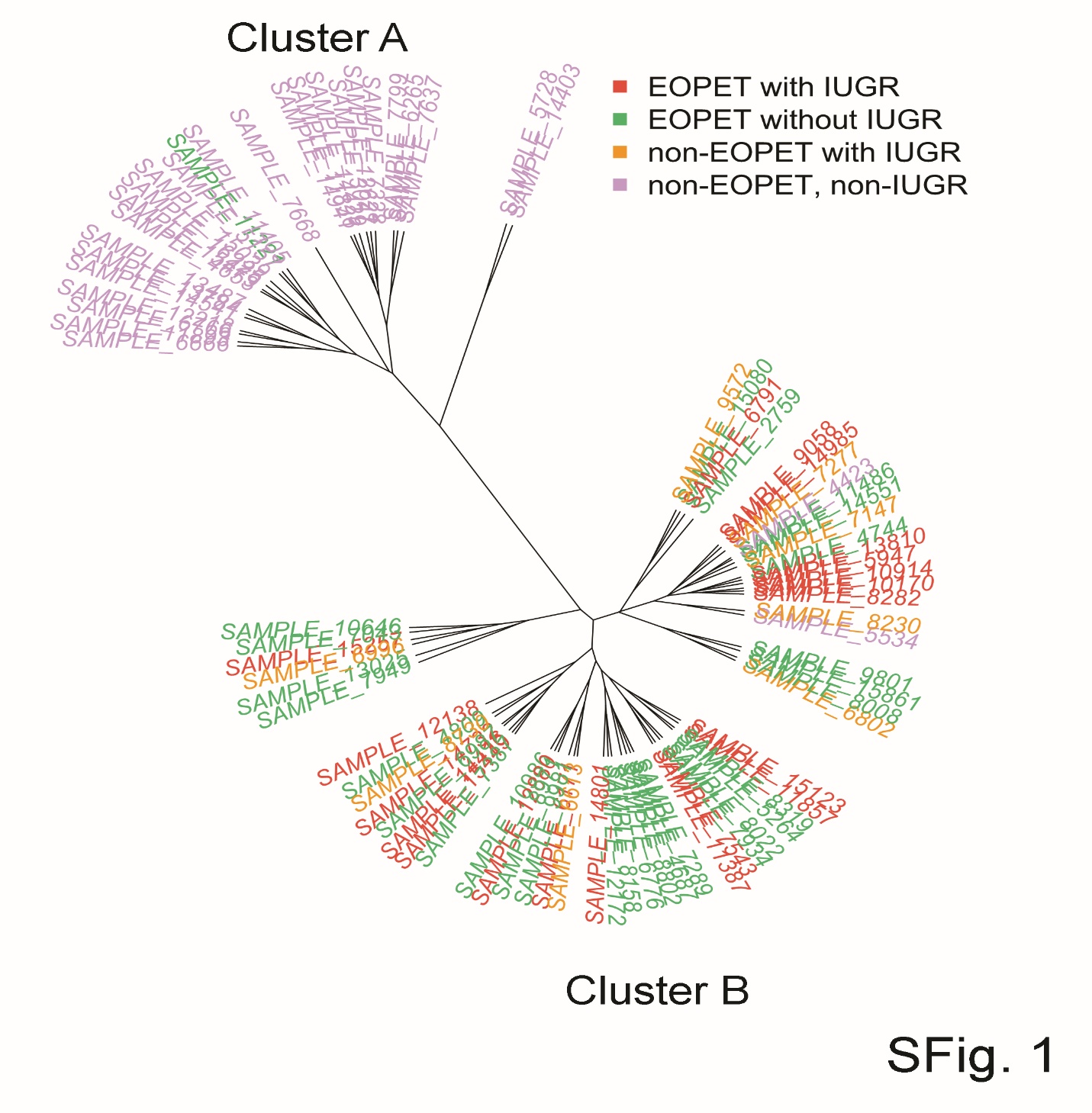


**Supplementary Fig. 1 Hierarchical clustering of patients in the GSE75010 dataset using 17 differentially expressed genes associated with the TGF-β pathway**. Clustered samples were colored by EOPE with IUGR (n=20), EOPE without IUGR (n=29), non-EOPE with IUGR (n=8), non-EOPE and non-IUGR (n=27).


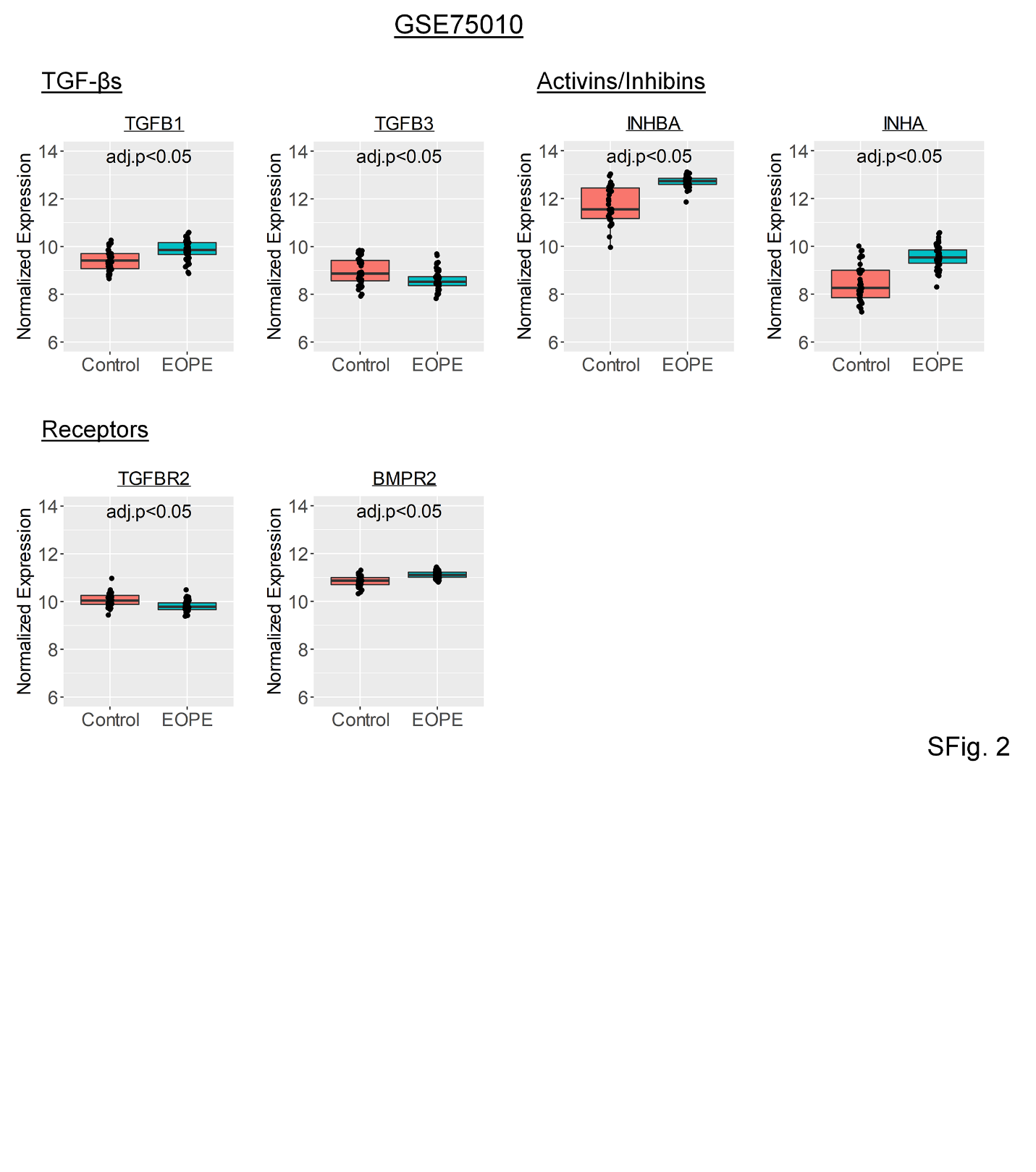


**Supplementary Fig. 2 TGF-β pathway-associated genes expression in healthy and EOPE placentas.** The expression levels of TGF-β superfamily ligands and receptors that were identified differentially expressed in GSE75010 dataset in placental tissues from EOPET *vs.* matched controls. Expression values were represented as log2 expression microarray data.


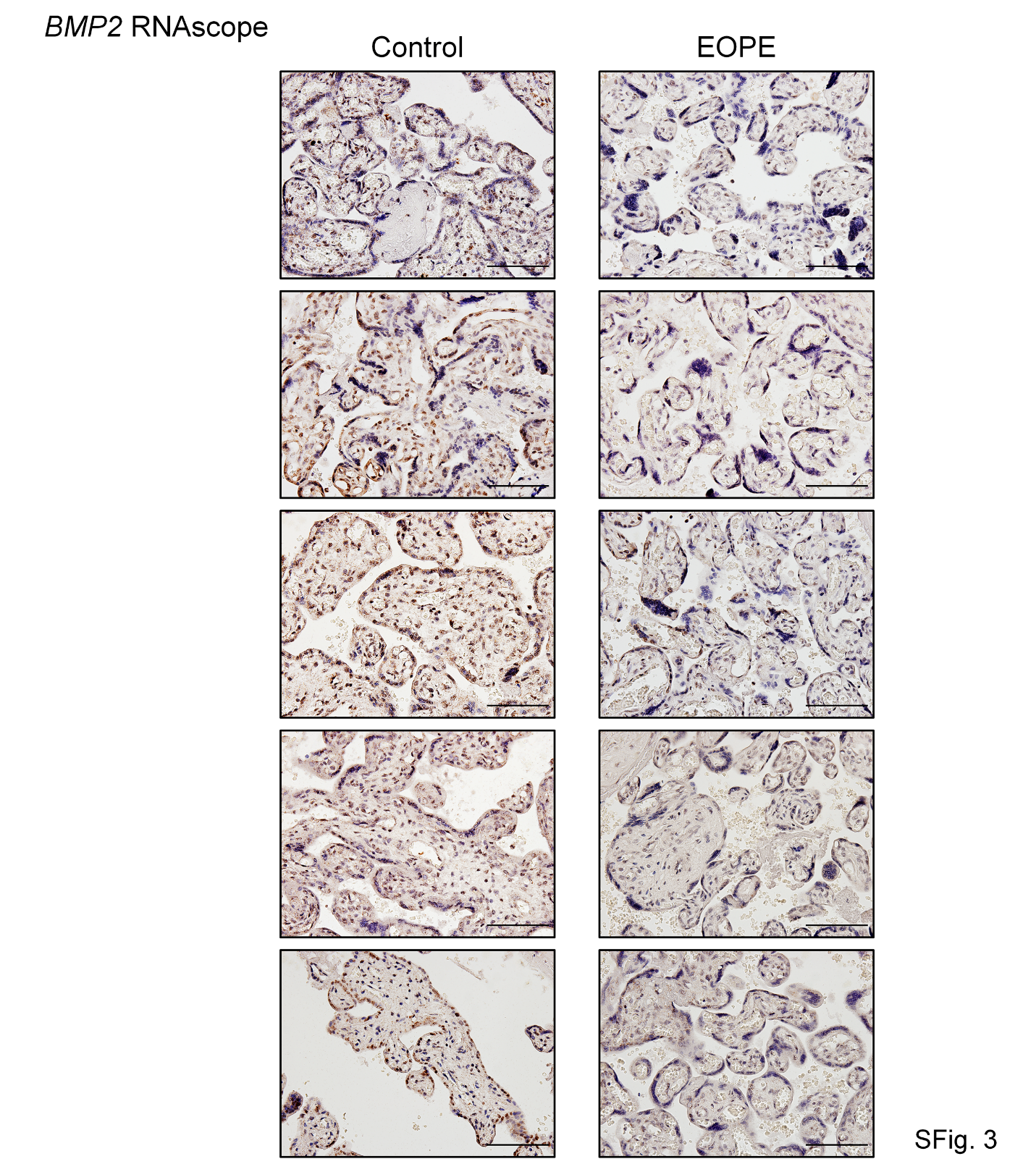


**Supplementary Fig. 3 BMP2 is aberrantly expressed in EOPE placenta.** Representative images showing *in situ* localization of *BMP2* mRNA transcript (brown) in control and EOPE placentas (200×). Scale bars = 100 µm.


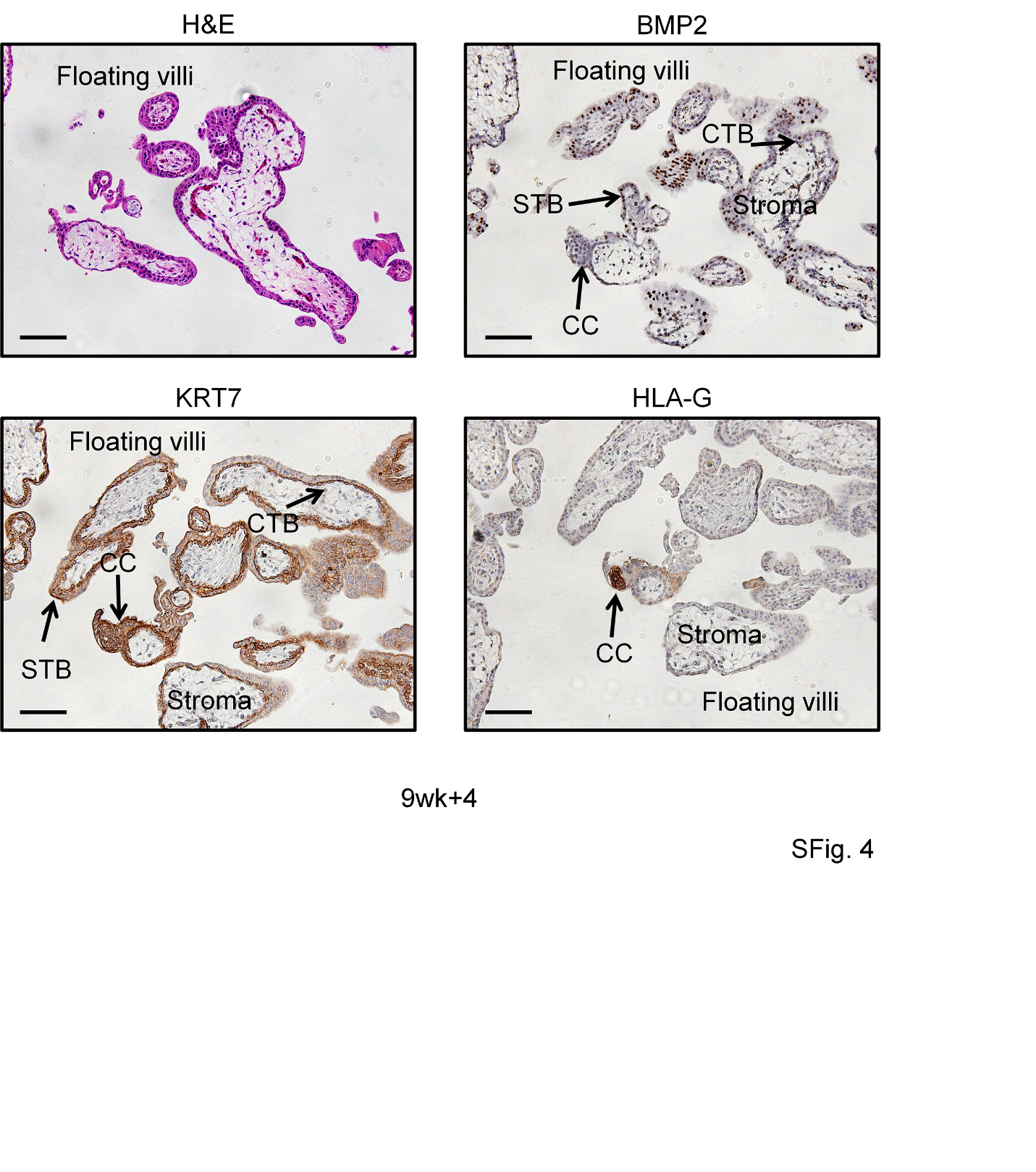


**Supplementary Fig. 4 BMP2 expression in human first-trimester placental floating villi.** Hematoxylin and eosin (H&E) staining (*top left*), *BMP2* mRNA RNAscope *in situ* hybridization (*top right*), and immunohistochemical staining for cytokeratin 7 (KRT7; *bottom left*) and human leukocyte antigen-G (HLA-G; *bottom right*) was performed on serial sections of the same placental villi (9 weeks + 4 days gestation; 100× magnification, scale bars = 100 µm). CTB, cytotrophoblast; SCT, syncytiotrophoblast; CC; anchoring cell column.


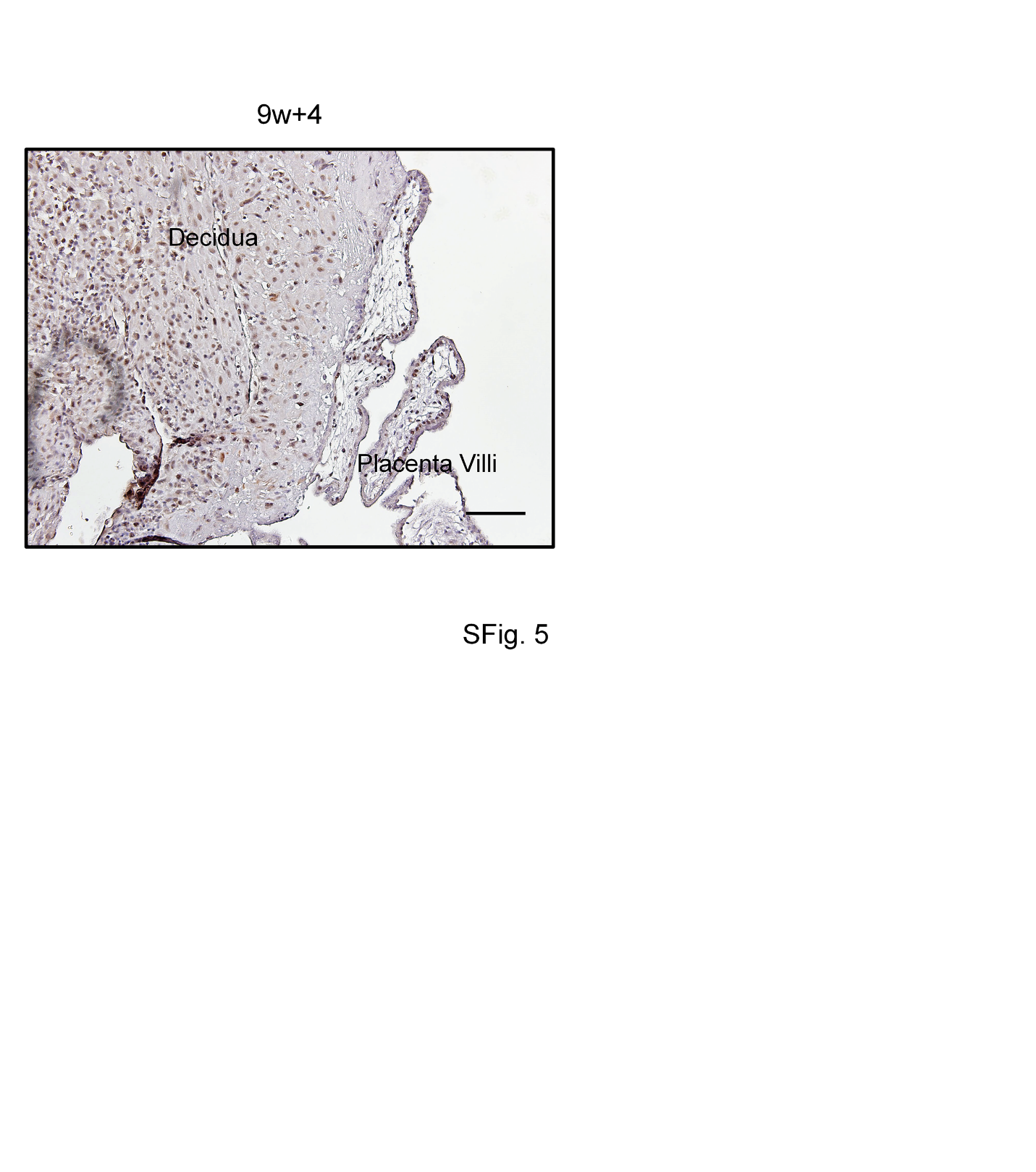


**Supplementary Fig. 5 Expression of AMIGO2 in human placental villi and decidua tissue.** AMIGO2 Representative immunohistochemical image showing AMIGO2 localization (brown) within first-trimester placental villus and decidua (9 weeks + 4 days gestation). 100 × magnification, scale bar = 100 µm.


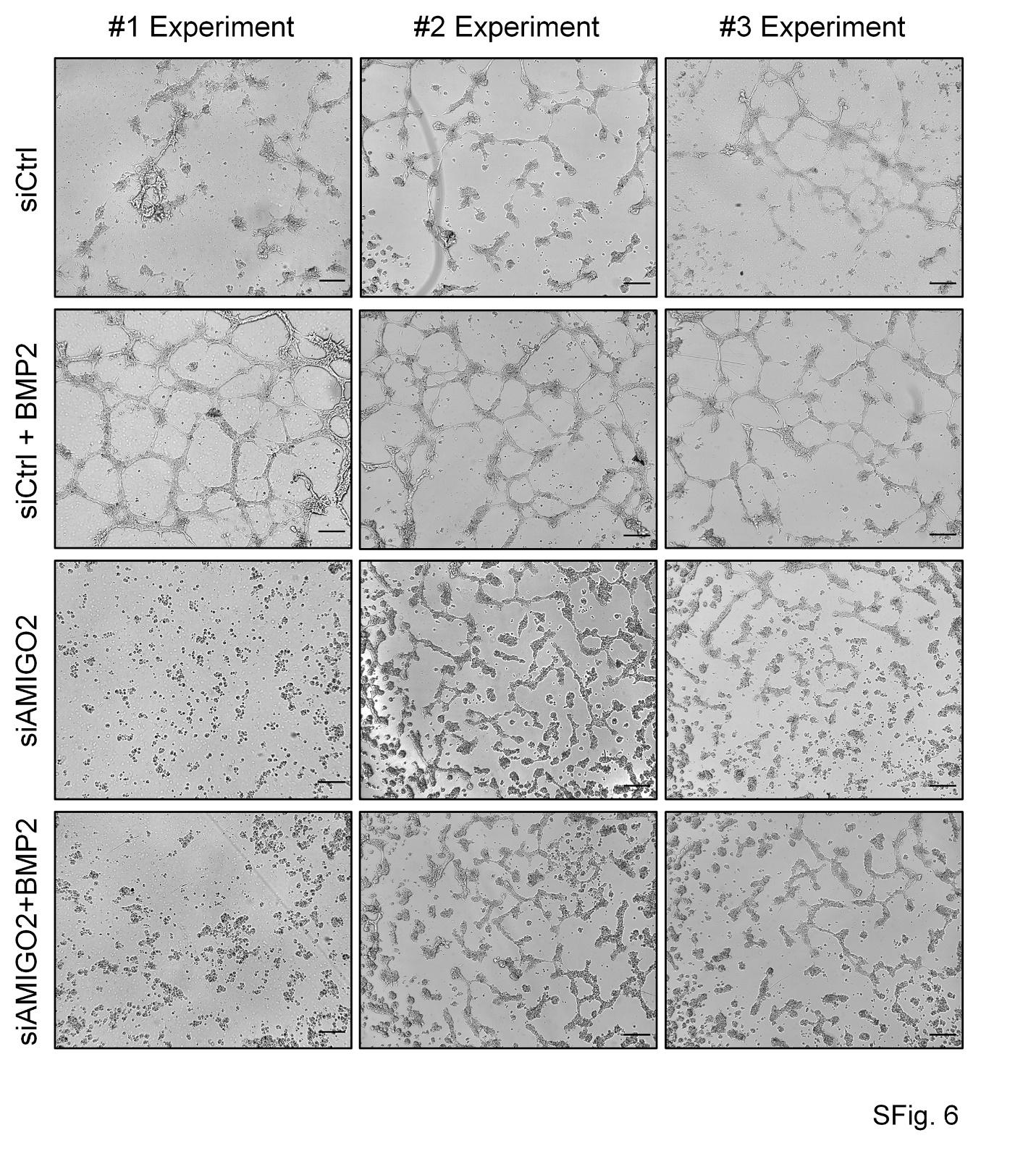


**Supplementary Fig. 6 AMIGO2 mediates BMP2-induced human endothelial-like tube formation.** Representative images of the effect of AMIGO2 knockdown on BMP2-induced endothelial-like tube formation in HTR-8/SVneo cells from three independent experiments.
